# Rhizocompartments drive the structure of root-associated fungal communities in halophytes with different life forms

**DOI:** 10.3389/fpls.2025.1584398

**Published:** 2025-06-03

**Authors:** Zhengxian Mo, Hengfang Wang, Li Sun, Yabei Zhang, Shengtao Wei, Hao Huang

**Affiliations:** ^1^ College of Ecology and Environment, Xinjiang University, Urumqi, China; ^2^ Key Laboratory of Oasis Ecology of Ministry of Education, Xinjiang University, Urumqi, China

**Keywords:** community composition, co-occurrence network, rhizocompartments, life forms, halophytes

## Abstract

**Introduction:**

Symbiotic fungi with plants are important for plant nutrient uptake and resource redistribution.

**Methods:**

High-throughput sequencing was used to investigate the composition and driving factors of fungal communities in three rhizocompartments (root endosphere, rhizosphere soil, and non-rhizosphere soil) of different halophyte life forms in the National Nature Reserve of Ebinur Lake Wetland in Xinjiang, China.

**Results:**

(1) The α-diversity index differed significantly among the three rhizocompartments of halophytes with different life forms (*P* < 0.05), and α and β-diversity were mainly driven by rhizocompartments. (2) Ascomycota and Basidiomycota were the dominant communities across various rhizocompartments in the different life forms. *Aporospora* and *Monosporascus* were the dominant fungal genera in the root endosphere of all three plant life forms. *Alternaria* was dominant in both rhizosphere and non-rhizosphere soils in herb. *Penicillium* and *Knufia* were the dominant in the rhizosphere and non-rhizosphere soils in shrub, respectively. While *Penicillium* and *Aspergillus* were dominant in both rhizosphere and non-rhizosphere soils in abor. (3) The complexity of the fungal co-occurrence network varied among plant life forms; the highest complexity was found in the rhizosphere soil of herb (11.102), the root endosphere of shrub (23.837) and in the non-rhizosphere soil of arbor (9.920). Furthermore, the co-occurrence networks of the three plant life forms in the three rhizocompartments were mainly positively correlated (86.73%–97.98%). (4) Root-associated fungal communities were significantly and strongly correlated with soil and root water content, soil and root total nitrogen, root and leaf total phosphorus, alkaline phosphatase, nitrate nitrogen and salt content in herb. While in shrub, root-associated fungal communities were strongly correlated with soil water content, available phosphorus, catalase and total phosphorus. However, arbor exhibited no significant correlations with soil and plant physicochemical factors.

**Discussion:**

These results provide a theoretical foundation for understanding the complex interaction mechanism between desert halophytes and fungi and are of great significance for strengthening desert vegetation management and vegetation restoration in arid areas.

## 1 Introduction

Climate change is intensifying drought conditions in arid regions (Maestre et al., 2015), which has caused over 5 million km^2^ of dryland desertification (Burrell et al., 2020). Vegetation is essential for windbreaks and sand fixation. In the extreme desert environment, plants have evolved specific adaptation strategies to deal with drought stress (Du et al., 2024; Zhao et al., 2024). Plant life forms are ecologically adaptable in terms of their structure, function, and morphology that change over time as they adjust to their external environment (Raju et al., 2014). Plants differ in nutrient utilization strategies and ecological adaptability depending on life forms (Cheng et al., 2022). Various plant life forms exhibit differences in leaf nutrient uptake (Hu et al., 2023) and respond to environmental changes (Zhang et al., 2022). In addition, the interactions between the different life forms of plants and microorganisms are different. Study has shown that AM fungi combined with different plant life forms show certain host specificities (López-García et al., 2017). The functional characteristics of different life forms of plants and their responses to environmental changes have been extensively studied; however, the root microbes of desert halophytes, especially those with a variety of life forms, have received very little attention.

Soil microorganisms in the soil are crucial for the intricate relationships that develop between plant root systems and the soil (Mercado-Blanco et al., 2018). The adaptability of desert vegetation is significantly influenced by soil microorganisms (Mengual et al., 2014) that play crucial roles in enhancing plant resilience to disease and stress (de Zelicourt et al., 2013), nutrient provision (Thepbandit and Athinuwat, 2024), and plant hormone synthesis (Innerebner et al., 2011). Fungi are integral to the functionality of intricate ecosystems and play substantial roles in both plant and soil ecological processes (Wagg et al., 2019). In soil ecosystems, fungi exert a more significant influence on soil organic carbon turnover and the carbon cycle than bacteria (Husain et al., 2024). Furthermore, fungal communities exhibit greater resistance to abiotic environmental stresses than bacteria (Luo et al., 2023). For instance, under drought conditions, the bacterial network is less stable than the fungal network in the soil (de Vries et al., 2018), and resistance to drought is stronger (Gao et al., 2022). Therefore, exploring the plant-soil-fungi continuum will help to better understand how plants adapt to environments when symbiotically interacting with microorganisms.

Research on plant-associated microorganisms has increasingly focused on the diversity of the rhizosphere microorganisms. Plant root activity affects the diversity and structure of microorganisms found in different parts of the roots, creating a unique rhizocompartmental niche in the non-rhizosphere soil, rhizosphere soil, and root endosphere (Edwards et al., 2015; Zhuang et al., 2020). Plants in different rhizocompartmental niches can recruit specific groups of microbes with distinct functions and adaptability (Foster et al., 2017). Study has shown that rhizocompartments produce the strongest niche differentiation between the rhizosphere soil and root endosphere communities (Chen et al., 2020) and that each rhizocompartment has a unique microbial community (Edwards et al., 2015). Nevertheless, current research on microorganisms in various rhizocompartments have predominantly focused on specific plant species, with a significant lack of understanding of the niche differentiation of microbial rhizocompartments across diverse plant life forms.

The Ebinur Lake Basin, located in western China, is part of an arid and ecologically fragile area. Soil salinization is a serious problem, and saline soil breeds a variety of halophytes (Xie et al., 2009). The soil microorganisms in the Ebinur Lake Basin, especially the composition and assembly mechanisms of soil microbial communities and the variables that influence them, have been studied (Wu et al., 2023; Yang et al., 2023). Previous studies have examined the composition of the soil bacterial communities and how they react to environmental factors (Zhao et al., 2018; He et al., 2021). Moreover, studies have focused on specific flora, such as denitrifying bacteria (Niu et al., 2022), nitrogen-fixing bacteria (Zhang et al., 2021), ammonia-oxidizing bacteria (He et al., 2017), and myxobacteria (Chen et al., 2023). Previous studies have focused on the composition and diversity of fungal communities and the variables that influence them (Ding et al., 2023; Qi et al., 2023). Nevertheless, the majority of previous studies on fungi were either limited to a certain plant or did not focus on rhizocompartments (root endosphere, rhizosphere soil and non-rhizosphere soil).

Therefore, we selected six halophytes (three life forms) from the Ebinur Lake Wetland National Nature Reserve (ELW) and analyzed the composition and driving factors of fungal communities in the root endosphere, rhizosphere soil, and non-rhizosphere soil. This study aimed to explore the following scientific issues: (1) how the composition and co-occurrence network patterns of fungal communities in different rhizocompartments of halophytes in three life forms are influenced by rhizocompartments and host life forms, and (2) how biotic (root and leaf water content, leaf and root organic carbon, etc.) and abiotic factors (soil salt content, soil water content, pH, etc.) influence the composition of fungal communities. This study helps to characterize the interactions between plant and microbial communities and provides a theoretical basis for understanding the symbiotic relationship between root systems and fungal communities in desert halophytes with different life forms.

## 2 Materials and methods

### 2.1 Overview of the study area

The study area is located on the northern bank of the Aqikesu River in the Ebinur Lake Basin (44°30’ - 45°09’N, 82°36’ - 83°50’E). The region has the typical characteristics of a temperate continental arid climate, with scarce precipitation (annual precipitation < 150 mm) and intense evaporation (annual evaporation capacity > 2,000 mm). The soil texture was coarse, with sandy soil and sandy loam being the main soil types. Soil salinization in this area is relatively serious, and most plants are halophytes, including *Populus euphratica*, *Haloxylon ammodendron*, *Nitraria tangutorum*, *Reaumuria songonica*, *Suaeda salsa*, *Salsola collina*, and *Alhagi* sp*arsifolia* (Xie et al., 2009).

**Figure 1:**
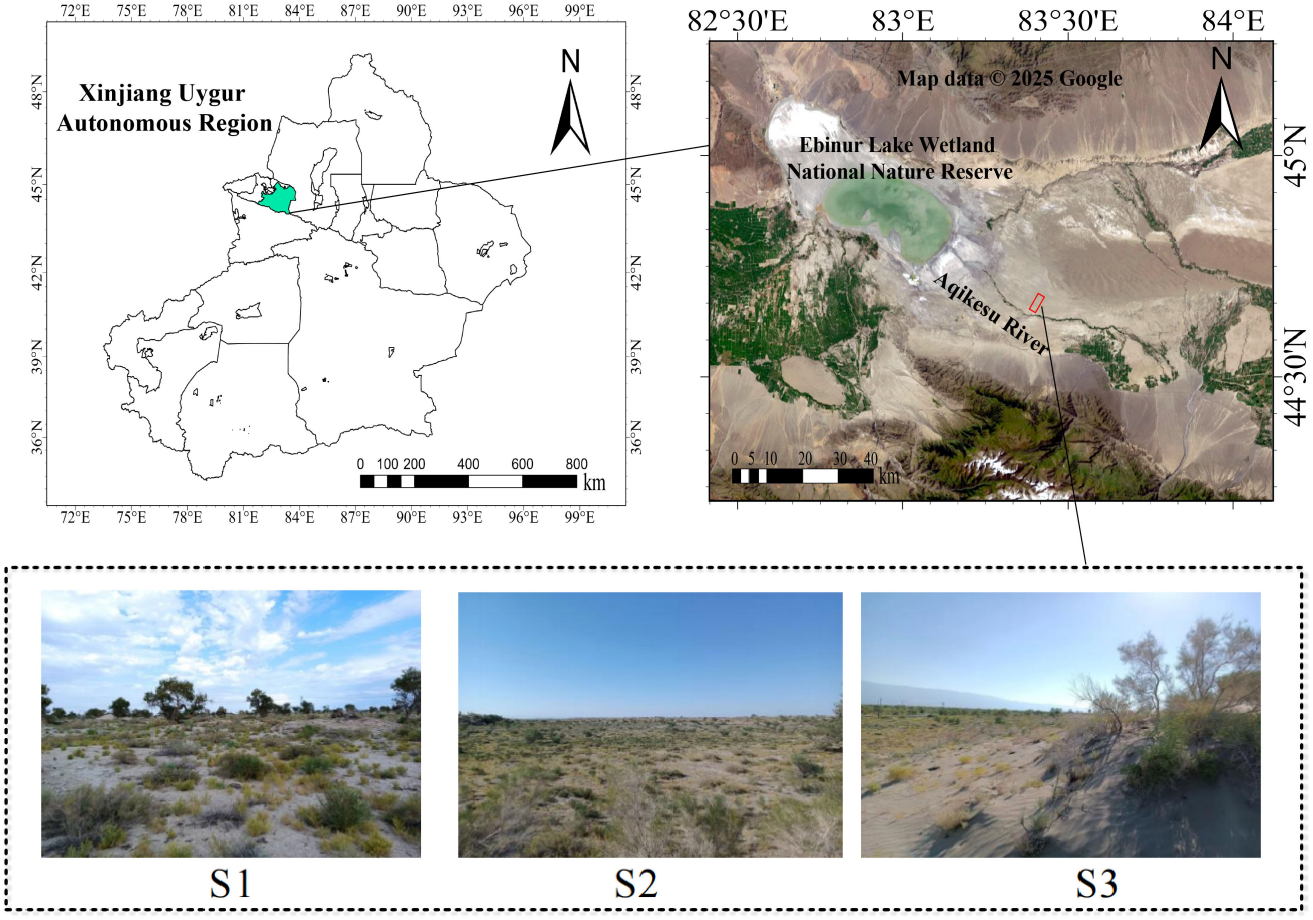
The study area’s location and sample layout. S1, S2, and S3 are field photographs of the three sample plots.

In the summer (August) of 2023, three 20 × 20 m sample quadrats were established at equal distances of approximately 500 m along the vertical riverbank direction (**Figure 1**). Each quadrat sample included six halophytes of three life forms: herb (*S. salsa* and *S. collina*), shrub (*N. tangutorum* and *R. songarica*), and arbor (*P. euphratica* and *H. ammodendron*). Six well-grown plants were chosen from each sample quadrat, and three replicates of each plant were collected, totaling 54 plants.

### 2.2 Sample gathering

Herbaceous plants were dug out whole with soil. Root samples of shrubs and arbors were collected from the fine root concentrated distribution area at a depth of 0–20 cm of soil around the trunk. The samples were subpackaged, marked, collected in sterile bags, and immediately refrigerated and sealed. Two root samples were extracted from each of the 54 plants, for a total of 108 fine root samples. Of these, 54 samples were used to determine the root physicochemical indices, and 54 were used for DNA extraction. At the same time, a relatively complete leaf from each plant was collected and stored to determine the plant leaf physicochemical indices. The 54 fine root samples collected for DNA extraction were separated into three parts: non-rhizosphere soil, rhizosphere soil, and root endosphere. Non-rhizosphere soil was collected directly shaken off from the roots (Zhuang et al., 2020). Rhizosphere soil (approximately 1 mm thick around the roots) was defined as the portion that could not be removed by direct shaking (Edwards et al., 2015). Rhizosphere soil and root endosphere samples were obtained as described by Bulgarelli et al. (2012). Fine roots with attached rhizosphere soil were placed into a 50 mL sterile centrifuge tube, and 10 mL of sterilized 10 mM PBS buffer was added. The roots were washed on a shaker at 200 rpm for 30 min. Roots were transferred to a new 50 mL sterile centrifuge tube, 10 mL PBS buffer was added, and the tubes were washed again (30 min, 200 rpm) to clean all the soil from the root surfaces. All washing buffers were combined and subjected to centrifugation (1500 × g, 20 min) and the remaining soil pellets were defined as the rhizosphere samples. After the second wash, the roots were transferred to another sterile centrifuge tube, and 10 mL of PBS buffer was added for ultrasonic treatment (160 W, 60 s on and 30 s off, for 10 cycles). The PBS buffer was then removed by centrifugation, and the washed and sonicated roots were defined as the root endosphere samples. Finally, we obtained 54 root endosphere samples, 54 non-rhizosphere soil samples, and 54 rhizosphere soil samples, and these 162 samples were stored at -80 °C for DNA sequencing.

### 2.3 Determination of soil and plant physicochemical indices

Non-rhizosphere soil was air dried for soil physicochemical parameter determination, including soil water content (SWC), soil pH (pH), total nitrogen (TN), available phosphorus (AP), ammonium nitrogen (AN), alkaline phosphatase (ALP), total phosphorus (TP), nitrate nitrogen (NN), soil salt content (SC), soil organic carbon content (SOC) content, catalase activity (CAT) and urease (Ure) (Bao, 2020). The detailed measurement methods are provided in **Supplementary Table S1**. In subsequent analyses and calculations, the physicochemical values of the soil physical and chemical properties of the non-rhizosphere soil were used as the shared values of the root endosphere, rhizosphere soil, and non-rhizosphere soil.

Physicochemical parameters of the plants were determined using 54 leaf and fine root samples. The measurement indexes included root water content (GSWC), leaf water content (LSWC), leaf membrane stability index (MSI), leaf pH (PpH), leaf total phosphorus (LTP), leaf organic carbon content (LSOC), leaf total nitrogen (LTN), root total nitrogen (GTN), root total phosphorus (GTP), root organic matter (GSOM), leaf organic matter (LSOM), and root organic carbon content (GSOC) (Perez-Harguindeguy et al., 2013; Bao, 2020). The detailed measurement methods are listed in **Supplementary Table S2**.

### 2.4 DNA extraction and PCR amplification

The total DNA of the microbial community was extracted using the E.Z.N.A.^®^ soil DNA Kit (Omega Bio-Tek, Norcross, GA, U.S.) according to the manufacturer’s instructions. Fungal PCR amplification was performed using primers ITS1F (5′-CTTGGTCATTTAGAGGAAGTAA-3′) (Gardes and Bruns, 1993) and ITS2 (5′-CTGCGTTCTTCATCGATGC-3′) (White et al., 1990). Sequencing was performed using the Illumina NextSeq2000 platform (Shanghai Majorbio Bio-Pharm Technology Co., Ltd.). The sequences were clustered into Operational Taxonomic Units (OTUs) at a 97% similarity threshold, and chimeras were removed using UPARSE 7.1 (http://drive5.com/uparse/, version 7.1). To minimize the impact of sequencing depth and rare OTUs on subsequent alpha and beta diversity analyses, all sample sequence counts were rarefied to the minimum sample sequence count. The rarefied OTUs were taxonomically annotated by alignment against the UNITE 8.0 fungal database (http://unite.ut.ee/index.php, version 8.0) using the RDP classifier (http://rdp.cme.msu.edu/, version 2.11) with a confidence threshold of 70%. FUNGuild (http://www.funguild.org/, version 1.0) software was used to predict fungal fungi. The raw data were deposited in the National Center for Biotechnology Information (NCBI) SRA database under Bioproject number PRJNA1046269.

### 2.5 Statistical methods

Based on the OTUs level, alpha diversity indices, including the Sobs index, Shannon index, Simpson index, and Chao index, were calculated using Mothur v1.30.1 (http://www.mothur.org/wiki/Calculators). The differences in the α-diversity indices among fungal communities in the root endosphere, rhizosphere soil, and non-rhizosphere soil were tested for multiple independent samples using the Kruskal-Wallis rank sum test and Dunn’s test. Principal Coordinates analysis (PCoA) based on the Bray-Curtis distance was performed using the Vegan package (version 3.3.1), and permutational multivariate analysis of variance (PERMANOVA) was implemented using the Adonis function with 999 random permutations to analyze the differences in fungal community structure between groups. PERMANOVA was used to assess the percentage of variation explained by the treatment and its statistical significance, using the Vegan package (version 2.4.3). Linear discriminant analysis (LDA) effect size (LEfSe) (http://huttenhower.sph.harvard.edu/LEfSe) was performed to identify the significantly abundant taxa (phylum to species) of fungi among the different groups (LDA score > 4, *P* < 0.05). The primary factors influencing fungal community composition in the three rhizocompartments of different plant life forms were analyzed using the Mantel test. Data analysis and visualization were performed using the online platform of Majorbio Cloud Platform (Ren et al., 2022).

Furthermore, SPSS 27 (IBM Corporation, Armonk, NY, USA) was used to analyze the physical and chemical factors of the soil and plants of different life forms using one-way analysis of variance, and the LSD multiple comparison method was used to detect the differences between the indices of different life forms. Network structures of co-occurrence among the top 100 most abundant fungal genera at the genus level were analyzed in the three rhizocompartments of the three types of life forms. We constructed co-occurrence networks for fungal communities in various rhizocompartments of different plant life forms using Spearman correlations between genera, with thresholds set at |r| ≥ 0.5 and *P* < 0.05. We used Gephi (v 0.10.1) (https://gephi.org/) to visualize the co-occurrence networks and calculate the relevant topological parameters. The node size was set by complexity, the node was colored according to the phylum level, and the edge color was set by positive and negative correlations.

## 3 Results

### 3.1 α-diversity of fungal communities in the three rhizocompartments of plants with different life forms

High-throughput sequencing was conducted on the root-associated fungal communities of six halophytes with different life forms, generating a total of 6,897,356 raw sequences. After rarefying the sample sequences according to the minimum number of sample sequences, 3,687,048 high-quality reads were retained. The rarefaction curves calculated using the Sobs index for most samples indicated that the OTU curves reached a plateau (**Supplementary Figure S1**), suggesting sufficient sequencing depth and reliable data. OTU statistical analysis based on data rarefied to the minimum sample sequence count revealed that among plants with three life forms, the number of OTUs was the highest in arbor and the lowest in shrub (**Table 1**). The number of OTUs in each rhizocompartment differed among the different life forms. Overall, the number of OTUs was the highest in the rhizosphere soil and the lowest in the root endosphere (**Table 1**).

**Table 1:** Number of OTUs and sequences in three rhizocompartments of different life-form plants.

Groups	Herb		Shrub		Arbor		All
	*Salsola collina*	*Suaeda salsa*	*Reaumuria songarica*	*Nitraria tangutorum*	*Populus euphratica*	*Haloxylon ammodendron*	
Root endosphere							
OTU	439	542	334	409	492	562	2778
Sequences	456279	590649	493466	564176	501715	532174	3138459
Rhizosphere soil							
OTU	792	2048	1316	1551	1829	1949	9485
Sequences	153932	340189	392235	380452	529992	483152	2279952
Non-rhizosphere soil							
OTU	902	633	401	247	1309	598	4090
Sequences	197578	238024	233266	164062	427071	218944	1478945

**Figure 2:**
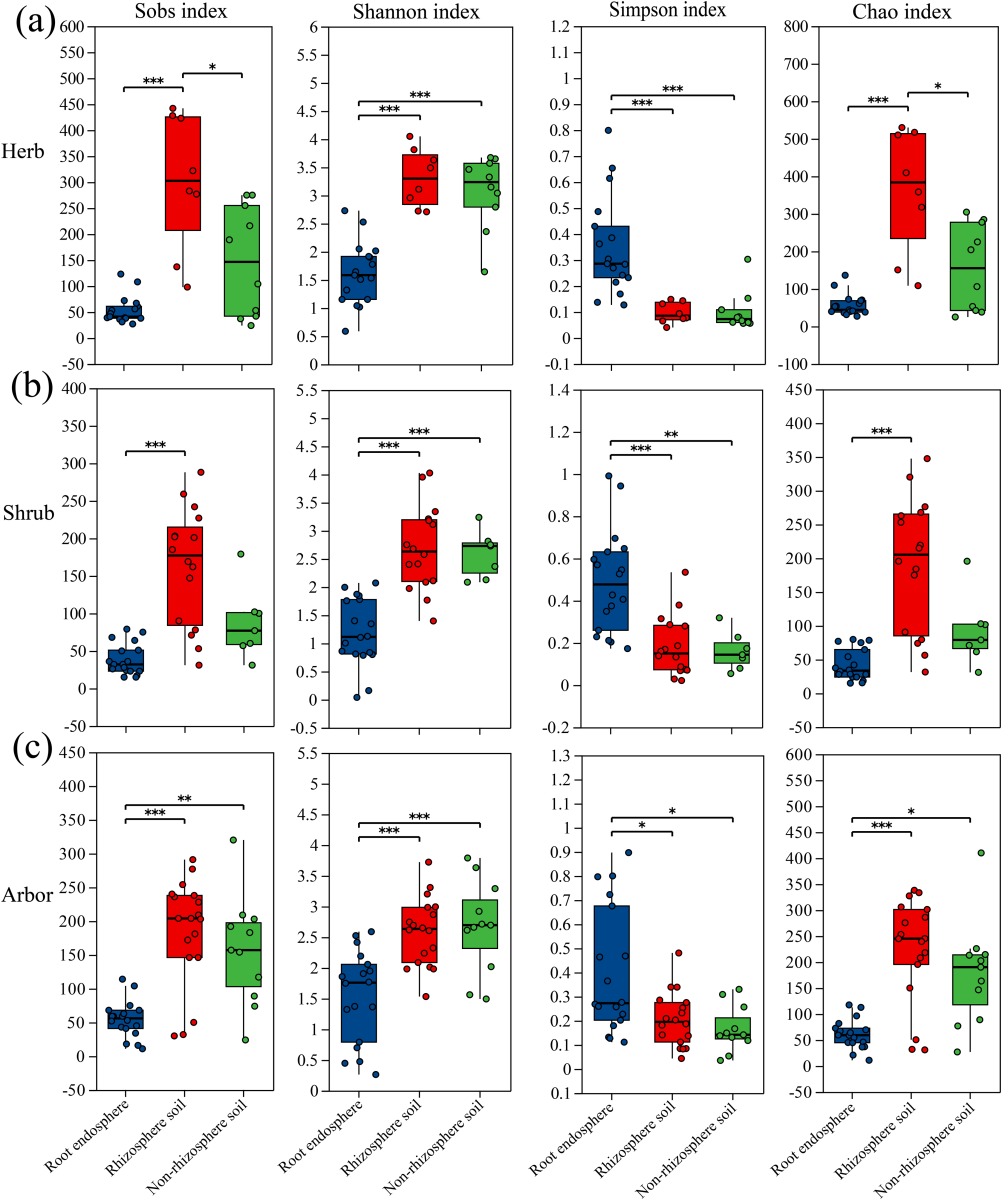
α diversity in three rhizocompartments of different life-form plants. **(a)** four diversity indices of herbaceous plant; **(b)** Four diversity indices of shrub plant; **(c)** Four diversity indices of arbor plant. Significance determined via Kruskal-Wallis rank-sum test followed by Dunn’s test for multiple comparisons, *P ≤ 0.05, **P ≤ 0.01, ***P ≤ 0.001.

The diversity indices showed significant variation among the various plant life forms found in the root endosphere, rhizosphere soil, and non-rhizosphere soil (**Figure 2**). Among the three life forms, the Shannon index in the root endosphere was significantly lower than that in the rhizosphere and non-rhizosphere soils, Furthermore, the Simpson index was significantly higher in the root endosphere than in the rhizosphere and non-rhizosphere soils. The Sobs and Chao indices revealed different patterns among the three life forms (**Figure 2**). Overall, in the three plant life forms, there were differences in diversity indices among the different rhizocompartments. In contrast, in different rhizocompartments, the diversity indices of fungal communities in herb, shrub and arbor were only different in the rhizosphere soil (**Supplementary Figure S2**). These findings indicate that the difference in α-diversity was primarily driven by the rhizocompartment and not by the plant life form.

### 3.2 β-diversity of fungal communities in the three rhizocompartments of plants with different life forms

**Figure 3:**
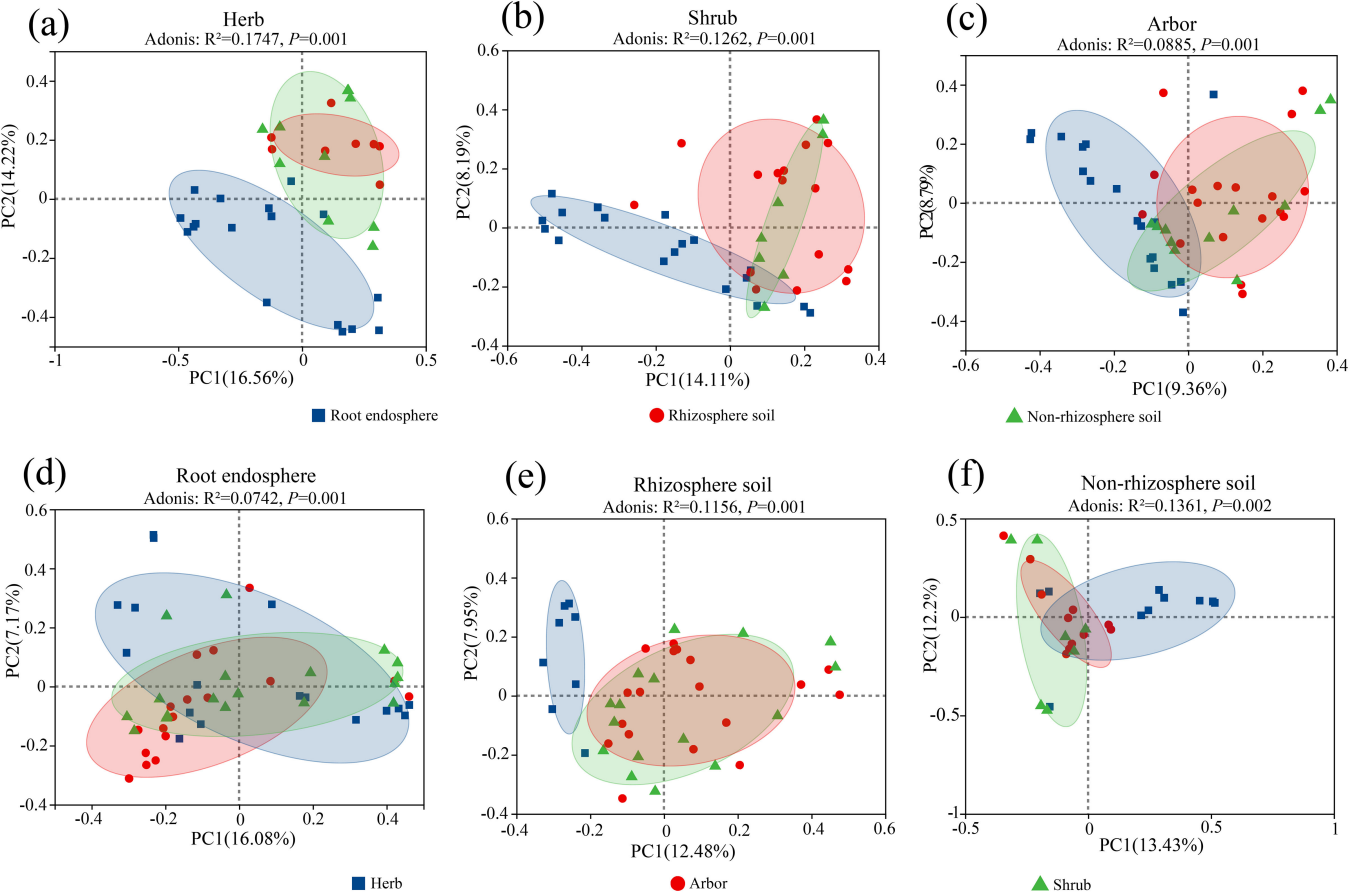
PCoA analysis of fungal community in different life forms and different rhizocompartments. **(a)** Herb; **(b)** Shrub; **(c)** Arbor; **(d)** Root endosphere; **(e)** Rhizosphere soil; **(f)** Non-rhizosphere soil. PCoA analysis was performed at the OTU level using the Bray-Curtis distance metric. Adonis (PERMANOVA) analysis was used to assess the overall differences in microbial community structure between groups. For the analysis of group differences along the PC1/PC2 axes, the Wilcoxon rank-sum test was applied.

Principal coordinate analysis (PCoA) based on the Bray–Curtis distance showed that the composition of fungal communities differed across rhizocompartments and life forms (**Figure 3**). The root endosphere fungal communities of the three plant life forms were distinguishable from the rhizosphere and non-rhizosphere soil fungal communities. Among the three life forms, herb showed the highest cumulative explanatory rates for PC1 and PC2 (30.78%). Adonis analysis indicated that the rhizocompartment significantly affected the fungal community structure of herb (R² = 0.1747, *P* = 0.001), shrub (R² = 0.1262, *P* = 0.001), and abor (R² = 0.0885, *P* = 0.001) (**Figures 3a–c**). Among the three rhizocompartments, non-rhizosphere soil showed the highest cumulative explanatory rates for PC1 and PC2 (25.63%). Adonis analysis indicated that the life form significantly affected the fungal community structure of the root endosphere (R² = 0.0742, *P* = 0.001), rhizosphere soil (R² = 0.1156, *P* = 0.001), and non-rhizosphere soil (R² = 0.1361, *P* = 0.002) (**Figures 3d–f**). Overall, the results of the permutation multivariate analysis of variance showed that the rhizocompartment (R^2^ = 0.07771, *P* = 0.001) and life form (R^2^ = 0.05287, *P* = 0.001) had the highest contribution to difference in fungal community diversity (**Supplementary Table S3**). Therefore, the β-diversity in different rhizocompartments of different life forms was mainly driven by rhizocompartments.

**Figure 4:**
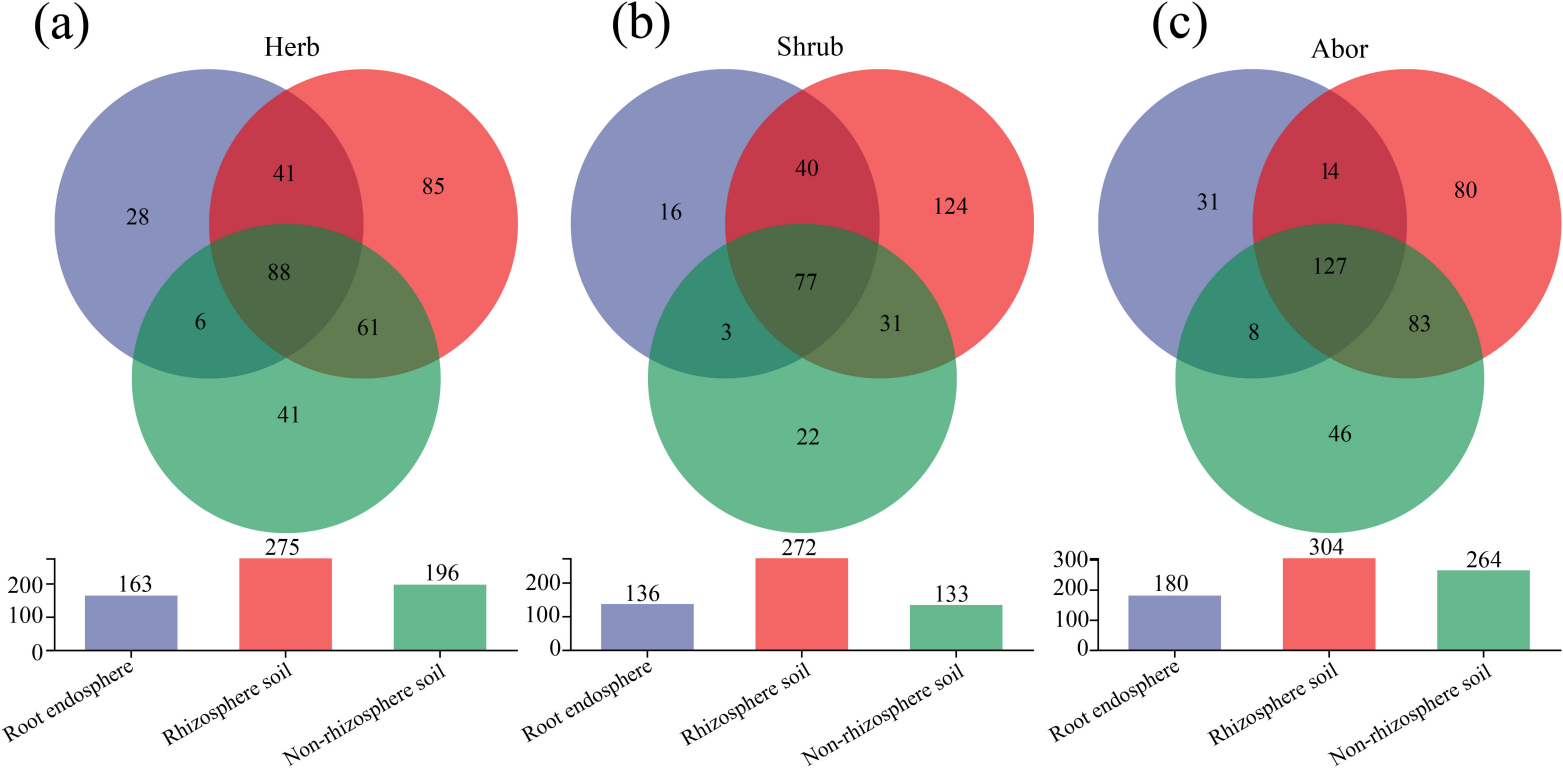
Venn diagram of fungal community in three rhizocompartments of different life forms at the genus level. **(a)** Herb; **(b)** Shrub; **(c)** Arbor. The vertical axis of the column chart represents the total number of species in each group at the genus level.

The Venn diagram illustrates 88 common genera among the three rhizocompartmental fungi in herb (**Figure 4a**). In shrub, there were 77 common genera among the three rhizocompartments (**Figure 4b**). In arbor, there were 127 common genera among the three rhizocompartments (**Figure 4c**). Among the three plant life forms, the number of endemic genera was the highest in the rhizosphere soil fungi and the lowest in the root endosphere.

### 3.3 Composition and biomarkers of the three rhizocompartments of plants with different life forms

**Figure 5:**
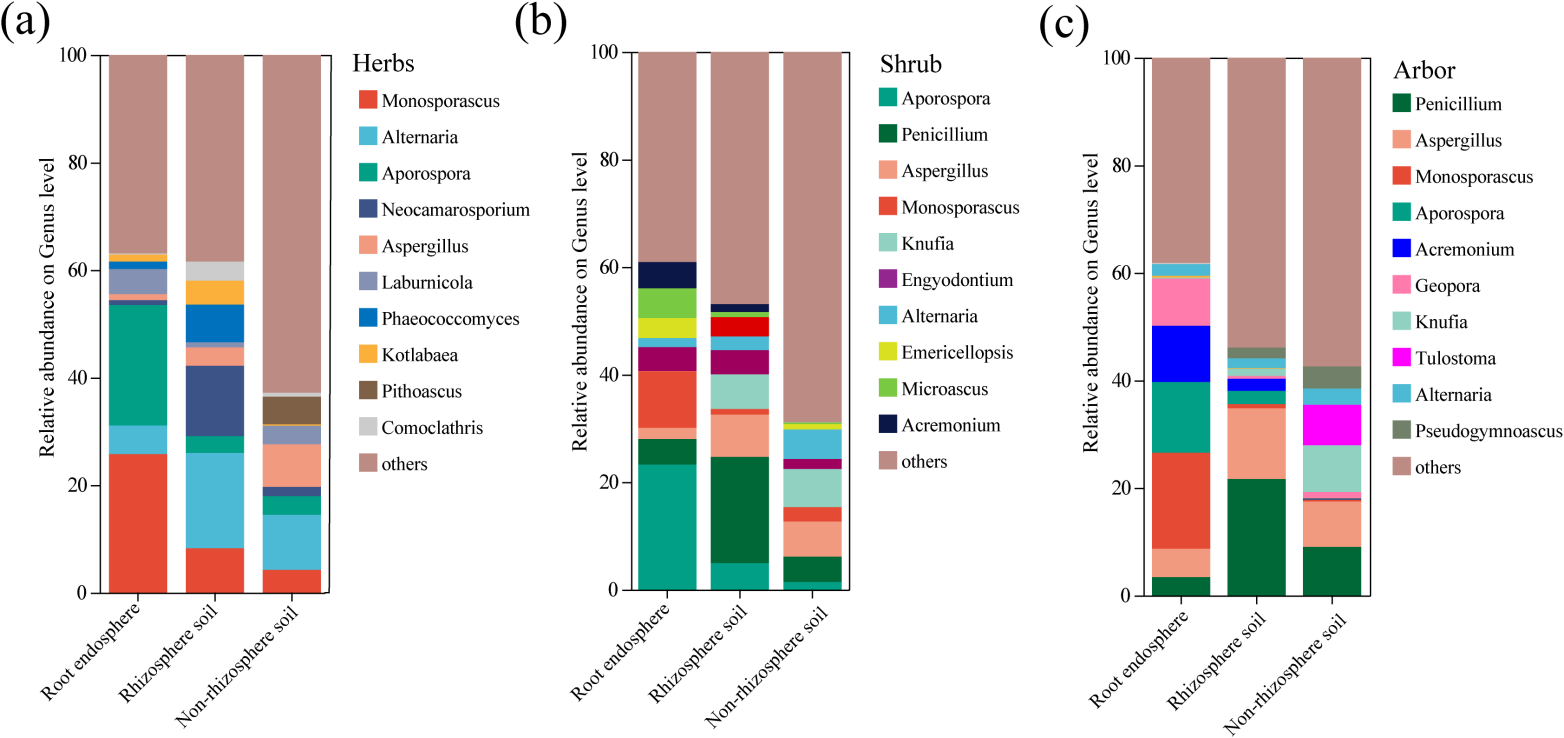
Comparison of fungal community relative abundance in different rhizocompartments with different life forms at the genus level. **(a)** Herb; **(b)** Shrub; **(c)** Arbor. The figure only shows the composition of the top 10 genera by abundance and their respective proportions, with other low-abundance genera and unclassified categories grouped as “others”.

The genus compositions of the three rhizocompartments of the different life forms were compared using community bar charts (**Figure 5**). In herb, *Aporospora* and *Monosporascus* were the dominant genera in the root endosphere, whereas *Alternaria* was the dominant genus in the rhizosphere and non-rhizosphere soils (**Figure 5a**). In shrub, *Aporospora and Monosporascus were* dominant in the root endosphere, *Penicillium* was dominant in the rhizosphere soil, and *Knufia* was dominant in the non-rhizosphere soil (**Figure 5b**). In arbor, *Aporospora and Monosporascus were* the main genera in root endosphere, while *Penicillium* and *Aspergillus* were dominant in the rhizosphere and non-rhizosphere soils (**Figure 5c**). The dominant genera in the root endosphere fungal community were distinct from those in the other two rhizocompartments in all the three plant life forms. Notably, *Aporospora* and *Monosporascus* were the dominant fungal genera in the root endosphere of all three plant life forms.

**Figure 6:**
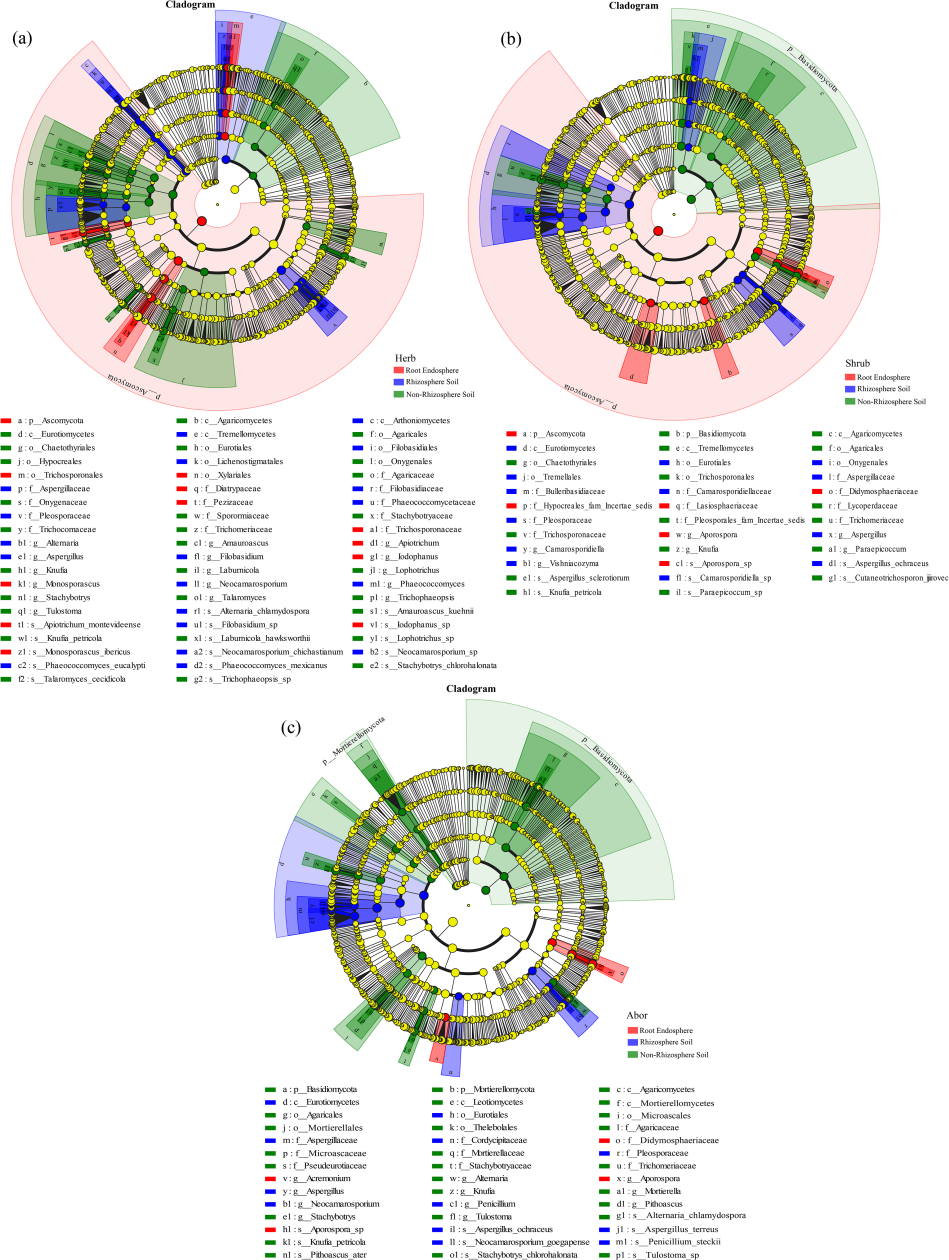
Evolutionary branch diagram of fungi in different rhizocompartments of three life-form plants. **(a)** Herb; **(b)** Shrub; **(c)** Arbor. Taxa with LDA > 4 and *P* < 0.05 were selected for analysis. The fungal taxa exhibiting significant differences across the three rhizocompartments are highlighted in red, blue and green dots, respectively, while yellow dots indicate taxa with no significant difference in abundance in each rhizocompartment.

LEfSe analysis revealed 59 fungal taxa (LDA > 4) with significantly different abundances among the three rhizocompartments in herb (**Figure 6a**, **Supplementary Figure S3a**). In the root endosphere, Diatrypaceae, Xylariales, and *Monosporascus* were significantly more abundant than in the rhizosphere and non-rhizosphere soils. In addition, Pleosporaceae and *Neocamarosporium* were considerably more abundant in the rhizosphere soil than in the other two compartments. The abundance of Eurotiomycetes was significantly higher in the rhizosphere soil than in the other two rhizocompartments (LDA > 5). In shrub, 35 fungal taxa (LDA > 4) showed significantly different abundances among the three rhizocompartments (**Figure 6b**, **Supplementary Figure S3b**). In the root endosphere, the abundance of Didymosphaeriaceae and *Aporospora* were significantly higher than in the other two rhizocompartments. In the rhizosphere soil, the abundance of Eurotiomycetes was notably higher than those in the other two rhizocompartments (LDA > 5). In arbor, 42 fungal taxa (LDA > 4) showed significantly different abundances among the three rhizocompartments (**Figure 6c**, **Supplementary Figure S3c**). The abundances of Didymosphaeriaceae and *Aporospora* were significantly higher in the root endosphere than in the other rhizocompartments. In contrast, the rhizosphere soil had much higher levels of Eurotiomycetes, Eurotiales, and Aspergillaceae than other rhizocompartments (LDA > 5). Among the three life forms, herb had the highest differential biomarkers.

Moreover, functional prediction analysis based on the FUNGuild database revealed the trophic composition characteristics and relative abundance distribution patterns of the fungal communities in the three rhizocompartments of the three plant life forms (**Supplementary Figure S4**). In herb and shrub, the root endosphere fungal community was dominated by the functional guild Endophyte-Lichen Parasite-Plant Pathogen-Undefined Saprotroph (**Supplementary Figure S4a** and **Supplementary Figure S4b**). In the rhizosphere and non-rhizosphere soils of both herb and shrub, the functional guild of Undefined Saprotroph was significantly enriched and had a relatively high relative abundance (**Supplementary Figure S4a** and **Supplementary Figure S4b**). In abor, the functional guild Undefined Saprotroph was dominant in all three rhizocompartments (**Supplementary Figure S4c**). In addition, the relative abundance of Plant Pathogen in the root endosphere fungal communities of herb and arbor was relatively high, and Animal-Pathogen-Endophyte-Plant Pathogen-Wood Saprotroph were abundant in the rhizosphere soil of herb (**Supplementary Figure S4**). Overall, fungal communities in the different rhizocompartments of plants with different life forms were primarily dominated by saprophytic fungi.

### 3.4 Fungal community co-occurrence network in the three rhizocompartments of plants with different life forms

**Figure 7:**
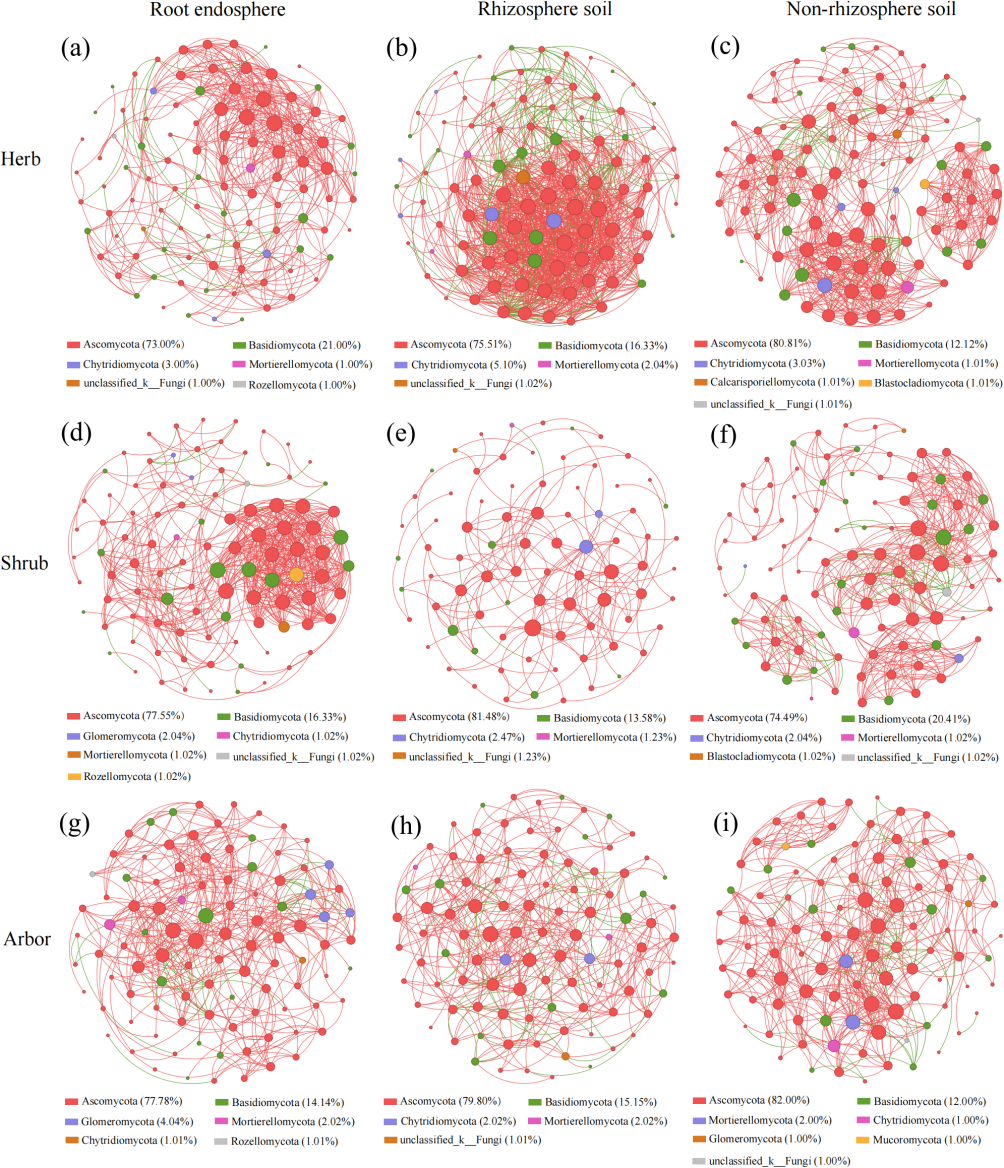
Co-occurrence network of fungi in different rhizocompartments of plants with different life forms. **(a–c)** represent the co-occurrence networks of root endosphere, rhizosphere soil, and non-rhizosphere soil in herb, respectively; **(d–f)** represent the co-occurrence networks of root endosphere, rhizosphere soil, and non-rhizosphere soil in shrub, respectively; **(g–i)** represent the co-occurrence networks of root endosphere, rhizosphere soil, and non-rhizosphere soil in abor, respectively. Nodes represented genera, node colors represented different phylums, and node size represented the average degree. Between any two nodes, a positive correlation was shown by a red line and negative by green.

The results of the co-occurrence network analysis suggested that the complexity of the fungal network was significantly influenced by the rhizocompartments and plant life forms (**Figure 7**, **Supplementary Table S4**). Overall, higher numbers of nodes, edges, and average degrees were displayed by fungal communities hosted in the rhizosphere of herb (98 nodes, 1168 edges, 23.837 average degree), the endosphere of shrub (98 nodes, 544 edges, 11.102 average degree), and the non-rhizosphere of arbor (100 nodes, 496 edges, 9.920 average degree) (**Supplementary Table S4**). Higher numbers of nodes, edges, and average degrees indicated that the microbial networks were more complex (**Figure 7**). The non-rhizosphere of herb (0.579) and shrub (0.636) and the root endosphere of arbor (0.512) had the highest modularity of fungal communities (**Supplementary Table S4**). The rhizosphere of herb (0.609, 2.295), the root endosphere of shrub (0.542, 3.092), and the non-rhizosphere of arbor (0.505, 2.793) have higher average clustering coefficients and shorter average path lengths, indicating that these fungal communities tended to form clusters and had a small-world network structure (**Supplementary Table S4**). Meanwhile, the co-occurrence networks of the three plant life forms in the three rhizocompartments were mainly positively correlated (86.73%–97.98%), and the most negative correlations were found in the rhizosphere soil of herb (13.27%) and arbor (12.18%) and the non-rhizosphere soil of shrub (10.37%) (**Figure 7**, **Supplementary Table S4**). Additionally, most of the nodes (genera) in the three rhizocompartments of different plant life forms belonged to Ascomycota (73.00%–82.00%) and Basidiomycota (12.00%–21.00%) (**Figure 7**).

### 3.5 Key factors influencing root-associated fungal communities in plants with different life forms

No significant differences were found in the soil parameters among plants with different life forms. The only significant difference found was in the ALP content between arbor and herb, with the latter having a much lower amount than the former (*P* < 0.05) (**Supplementary Table S5**). In terms of the physical and chemical characteristics of plants, LSWC, PpH, GTN, LSOC, and GSOC varied significantly (*P* < 0.05) (**Supplementary Table S5**).

**Figure 8:**
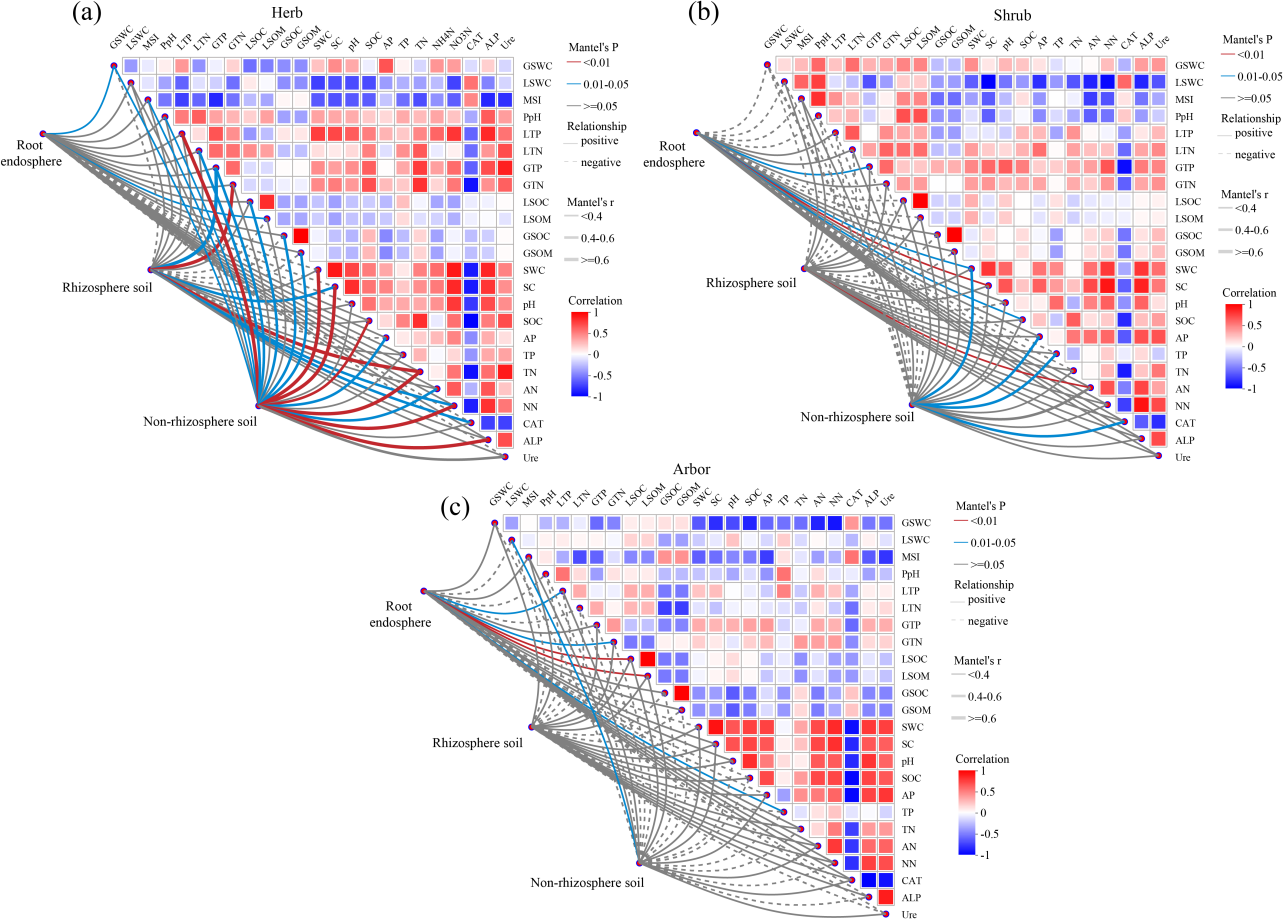
Correlation analysis of soil and plant factors influencing fungal communities in different rhizocompartments. **(a)** Herb; **(b)** Shrub; **(c)** Arbor. The line in the diagram represented the relationship between environmental and community aspects, while the heat map showed the correlations between environmental factors. Line thickness: the relationship between the community and the soil and plant parameters was determined using Mantel ‘r (| r |); Relationship: positive and negative represented the positive and negative correlation between the community and the soil and plant parameters. In the heatmap, different colors represent the positive and negative correlations between environmental factors, and the intensity of the colors represents the magnitude of the positive and negative correlations. SWC, water content; SC, salt content; pH, soil pH; SOC, organic carbon; AP, available phosphorus; TP, total phosphorus; TN, total nitrogen; AN, ammonium nitrogen; NN, nitrate nitrogen; CAT, catalase; ALP, alkaline phosphatase; Ure, urease; GSWC, root water content; LSWC, leaf water content; MSI, membrane stability index; PpH, leaf pH; LTP, leaf total phosphorus; LTN, leaf total nitrogen; GTP, root total phosphorus; GTN, root total nitrogen; GSOM, root organic matter; LSOM, leaf organic matter; GSOC, root organic carbon; LSOC, leaf organic carbon.

In herb, the composition of root endosphere fungal communities was weakly correlated with GSWC (r = 0.17, *P* = 0.049) and LSOM (r = 0.16, *P* = 0.047). Rhizosphere soil fungal communities were significantly and strongly correlated with GTN (r = 0.71, *P* = 0.001), GTP (r = 0.65, *P* = 0.016), and TN (r = 0.61, *P* = 0.008). Non-rhizosphere soil fungal communities were significantly and strongly correlated with LTP (r = 0.79, *P* = 0.001), ALP (r = 0.72, *P* = 0.005), NN (r = 0.68, *P* = 0.001), SWC (r = 0.66, *P* = 0.001), SC (r = 0.66, *P* = 0.007), and TN (r = 0.62, *P* = 0.004) (**Figure 8a**). In shrub, the composition of root endosphere fungal communities was moderately correlated with SC (r = 0.36, *P* = 0.005), GTP (r = 0.34, *P* = 0.012), and SOC (r = 0.31, *P* = 0.035). Rhizosphere soil fungal communities were weakly correlated with AN (r = 0.36, *P* = 0.005). Non-rhizosphere soil fungal communities moderately correlated with SWC (r = 0.54, *P* = 0.02), AP (r = 0.49, *P* = 0.02), CAT (r = 0.47, *P* = 0.034), and TP (r = 0.44, *P* = 0.042) (**Figure 8b**). In abor, the composition of root endosphere fungal communities was weakly correlated with LSOM (r = 0.36, *P* = 0.001), GTN (r = 0.25, *P* = 0.013), and TP (r = 0.20, *P* = 0.043). The non-rhizosphere soil fungal communities were weakly correlated with LSWC (r = 0.35, *P* = 0.028). However, the rhizosphere soil fungal communities did not correlated with any physicochemical factors (**Figure 8c**). Overall, non-rhizosphere fungal communities showed the strongest response to nutrients, followed by the rhizosphere soil, whereas the root endosphere showed the weakest response. Moreover, the intensity of this response decreased across plant life forms in the order herb > shrub > abor.

## 4 Discussion

### 4.1 Rhizocompartments drive root-associated fungal community composition

In this study, we analyzed the root-associated fungal communities of halophytes with three different life forms and found that the α diversity of the fungal community was significantly different among different rhizocompartments (root endosphere, rhizosphere soil, non-rhizosphere soil) (*P* < 0.05, **Figure 2**). Specifically, the fungal communities in the root endosphere of the three life forms showed the lowest richness, which is consistent with the results of Edwards et al. (2015) in rice and Zhuang et al. (2020) in mangroves. The results of PCoA further revealed that the β-diversity of root endosphere fungal communities was significantly different from that of rhizosphere and non-rhizosphere soil communities, and this pattern was consistent across multiple plant species (Zhou et al., 2023). Notably, the number of common fungal genera among the three rhizocompartments was relatively small, and the number of endemic genera in the root endosphere was also the lowest, indicating that the root system had a selective filtering effect on microorganisms (Yeoh et al., 2016). Some rhizosphere microbes can enter root tissue and form an endophytic microbiome, which may differ from the rhizosphere microbiome in terms of community structure (Lundberg et al., 2012), thus shaping a specific root compartment niche (Fitzpatrick et al., 2018). Overall, there was ecological niche differentiation in the fungal community composition between different rhizocompartments at the life-form level, which was mainly driven by the rhizocompartments.

Our study revealed that fungal communities across the rhizocompartments of different plant life forms were abundant in Ascomycota and Basidiomycota at the phylum level, with Ascomycota being the most abundant, which is consistent with previous reports on halophytic species (Li et al., 2021; Wang et al., 2025). As most fungi in Ascomycota and Basidiomycota are saprotrophic, their predominance in desert plants may enhance the *in situ* nutrient recovery potential and promote nutrient cycling in desert soils (Manici et al., 2024). The FUNGuild prediction results also supported this phenomenon, indicating that the fungal communities in different rhizocompartments in different life forms were predominantly composed of saprophytic fungi (**Supplementary Figure S4**). At the genus level, the dominant genera in different rhizocompartments of different life forms differed, and the dominant genera primarily belonged to Dothideomycetes (*Alternaria* and *Aporospora*), Eurotiomycetes (*Knufia*, *Aspergillus* and *Penicillium*), and Sordariomycetes (*Monosporascus*). The root endosphere, the core area for direct interactions between plants and microorganisms, has a significantly different fungal composition from that of the rhizosphere and non-rhizosphere soils. The dominant genera in the root endosphere of the three plant life forms were mainly *Aporospora* and *Monosporascus*, both of which have strong salt tolerance (Wang et al., 2022; Cavalcante et al., 2020). *Monosporascus* has been reported to maximize soil organic matter accumulation and cause soil acidification; its enrichment in the root endosphere can improve plant growth and ecological adaptability (Zuo et al., 2022). In the rhizosphere and non-rhizosphere soils, plants with different life form showed different dominant fungal genera. In herb, *Alternaria* dominates both rhizosphere and non-rhizosphere soils, and studies have shown that this genus promotes plant growth and enhances salt tolerance (Tang et al., 2021). In the rhizosphere and non-rhizosphere soil of shrub, the dominant genera were *Penicillium* and *Knufia*, respectively. *Penicillium* can promote plant growth by enhancing nitrogen mineralization (Osés-Pedraza et al., 2020), whereas *Knufia* not only shows significant adaptability to poor soil (Huang et al., 2024), but also has strong salt tolerance (Isola et al., 2022). In the rhizosphere and non-rhizosphere soils of abor, *Penicillium* and *Aspergillus* were the dominant genera. Fungi belonging to the genera *Aspergillus* and *Penicillium* can dissolve insoluble mineral phosphates in the soil, enhancing the availability of phosphorus in the soil (Khuna et al., 2021). In addition, certain fungal taxa exhibit trophic plasticity, functioning not only as saprotrophs involved in organic matter decomposition but also as plant pathogens that affect plant health (Chen et al., 2015). In specific ecosystems, plant pathogens may indirectly protect their hosts by competitively inhibiting stronger pathogens. For example, *Alternaria* can compete with *Fusarium* for the same habitat and inhibit its growth (Müller et al., 2015). Study has found that mycorrhizal fungi can reduce infection of host plant roots by pathogenic fungi and weaken the negative effects of pathogens by enhancing nutrient uptake and antagonizing pathogens (Liang et al., 2015). Overall, the composition of fungal communities reflects the adaptive traits of plants to environmental conditions, where plants with different life forms may host specific endophytes and rhizosphere-associated communities that facilitate their adaptation to saline desert environments.

The root-associated fungal communities of halophytes in different regions show significant differences and uniqueness. Compared to non-desert ecosystems, in the rhizosphere and non-rhizosphere soils of halophytes in the Yellow River Delta, *Scutellospora* is the dominant genus (Zhao et al., 2022), whereas in the endophytic fungal communities of halophytes growing on the coast of the western Aral Sea in Uzbekistan, the dominant genera are *Neocamarosporium*, *Botryosphaeria*, and *Alternaria* (Gao et al., 2023). Although there is some overlap in the taxonomic groups within the desert ecosystem, each region has unique characteristics. For instance, the common dominant genera of rhizosphere fungi in halophytes in Southern Xinjiang are *Fusarium*, *Acremonium*, *Aspergillus*, and *Penicillium* (Li et al., 2021), whereas in the endophytic fungal communities of halophytes in the West Ordos Desert, the dominant genera are *Fusarium*, *Dipodascus*, *Curvularia*, and *Penicillium* (Wang et al., 2025). These differences demonstrate that the regional environment has a crucial impact on the composition of fungal communities in halophytes and that the unique soil, climate, and other conditions in each area shape the unique symbiotic patterns between halophytes and fungi. Additionally, this study only investigated two species each of herb, abor, and shrub, which may limit the generalizability of the conclusions. Therefore, conducting multisite and multispecies studies in the future is necessary to verify the generalizability of the conclusions of this study.

### 4.2 Interactions of microorganisms in different rhizocompartments revealed by the co-occurrence network

The dynamics of the ecological niches of the microbial communities are reflected in the co-occurrence network, which shows the interactions between several genera (Li and Wu, 2018). According to the ecological network theory, the functionality of microbial communities and the stability of ecosystems are influenced by changes in co-occurring network relationships (de Vries et al., 2018). Co-occurrence networks play a significant role in shaping microbial community structure and regulating soil nutrient cycling efficiency (Harris, 2009). The dominance of positive correlations or reduction in negative correlations within microbial communities can disrupt community stability (Coyte et al., 2015), and a lower degree of modularity in high-stress environments may exacerbate these effects (Stein et al., 2013). Higher modularity in a network can help stabilize communities by confining the consequences of a taxon’s loss to its module, thus preventing the extinction of that taxon from affecting other parts of the network (Stein et al., 2013). Besides, it is believed that the stronger positive connections between nodes suggest the more synergistic interactions within microbial communities (Xiong et al., 2017), while negative correlations indicate competitive relationships in co-occurring networks (Zheng et al., 2017). Our research revealed that more than 86.73% of the fungal community’s co-occurrence networks displayed positive relationships between genera, whereas only a few showed negative relationships (**Figure 7**). This is consistent with previous studies, which found that communities dominated by positive correlations were more common in high-stress environments (Dong et al., 2024). This indicates that most root-associated fungi of different forms may adapt to the saline desert environment through synergistic rather than antagonistic interactions, which is in line with the results of other studies (Peng et al., 2023).

Furthermore, the number of nodes and edges of the network, as well as the network connectivity and complexity of the network in each compartment of the different plant life forms, varied. These findings are consistent with the co-occurrence network patterns of root-associated fungal communities across the three plant life forms reported by Liu et al. (2023). This may be related to the characteristics of different plant life forms, as this study found significant differences in LSWC, PpH, GTN, LSOC, and GSOC among the different plant life forms (**Supplementary Table S5**). Overall, we found that the co-occurrence network of herbaceous plants was the most complex. Another study has shown that network complexity is positively correlated with community stability (Guo et al., 2022), as complex networks enhance resilience to environmental changes and optimize resource transfer efficiency (Morriën et al., 2017). Therefore, the high connectivity and low modularity of the co-occurrence network in herbaceous plants may be closely related to their rapid lifecycle and dynamic root exudate secretion patterns.

### 4.3 Driving factors for differences in microbial communities in various rhizocompartments

In this study, we found that the composition of fungal communities in different rhizocompartments of plants is closely related to the physicochemical factors of the non-rhizosphere soil and the physicochemical factors of the plants. Soil factors are mainly related to the composition of the fungal communities in the rhizosphere and non-rhizosphere soils in different plant life forms, especially in herb and shrub (**Figure 8**). Soil is a nutrient substrate for direct microbial growth and reproduction and a major site for microbial activity; therefore, soil factors are strongly correlated with the composition of fungal communities in the rhizosphere and non-rhizosphere soils (Brown et al., 2020). Various soil characteristics influence plant growth and the metabolic traits of roots, thereby affecting the build-up of microorganisms associated with the roots (Qin et al., 2019). Plants exude large amounts of soil organic carbon and hormones from their root systems to attract microorganisms. This process modifies soil characteristics, which affect the rhizosphere microbial community (Bi et al., 2021). In arid desert ecosystems, soil moisture levels are crucial factors that restrict microbial activity (Armstrong et al., 2016). Studies have shown that water deficiency in the soil can hinder the absorption and transportation of nutrients by plants and intensify the competition for essential minerals between plants and surrounding microorganisms (Ouyang et al., 2016). Therefore, soil moisture affects the diversity and biomass of plant root-associated fungal communities (Maestre et al., 2015). Given the severe salinization in the ELW, where plants experience intense salt stress, soil salinity consequently emerges as a critical determinant in shaping root-associated fungal communities (Chandrasekaran et al., 2014). In summary, soil factors play important roles in the composition of root-associated fungal communities.

In this study, we found that factors influencing rhizocompartmental fungal communities differed among plants with different life forms. This was probably due to the combined influence of soil factors and vegetation type (Pan et al., 2021). The composition of root-associated fungal communities in different plant life forms was primarily related to plant physicochemical factors, indicating that plant genetic factors dominate fungal communities in the root endosphere (Lan et al., 2023). In addition, root-associated fungal communities were also correlated with soil factors, which is consistent with the results of previous studies (Peng et al., 2023). For example, the composition of the root endosphere fungal community of shrub is correlated with SC and SOC (**Figure 8b**). Notably, compared to abor and shrub, the composition of root-associated fungal communities in herbaceous plants was a closely related to soil and plant factors (**Figure 8**). This may be because root-associated fungal communities in herbaceous plants are sensitive to the soil environment and can respond rapidly to nutrient changes (Peng et al., 2023). In particular, the composition of fungal communities in the rhizosphere and non-rhizosphere in herbaceous plants had a significantly strong correlation with nitrogen and phosphorus nutrient indices (**Figure 8a**). This is probably because the levels of N and P in the soil significantly influence the distribution and activity of microbial communities in both soil and the root systems (Ren et al., 2021).

## 5 Conclusion

In this study, we explored the composition and co-occurrence network patterns of root-associated fungal communities in different halophyte life forms and discussed the main factors affecting community composition. We found that rhizocompartments and host life forms jointly influenced the α and β diversity of different rhizocompartmental fungal communities, but were mainly driven by rhizocompartments. Different life forms of halophytes adapt to the extreme desert environment through specific species compositions and community structures. This study revealed that the dominant genera in root-associated fungal communities of different plant life forms were mainly salt-tolerant or growth-promoting fungi. These fungi enhanced plant resistance, adaptability, and nutrient acquisition capacity, thereby promoting plant survival in extreme environments. In addition, root-associated fungal communities in halophytes with different life forms mainly adapt to the environment through positive cooperative relationships. Our findings provide a theoretical basis for understanding the interactions between desert halophytes with different life forms and fungi, as well as how desert halophytes adapt to extreme environments.

## Data Availability

The datasets presented in this study can be found in online repositories. The names of the repository/repositories and accession number(s) can be found below: https://www.ncbi.nlm.nih.gov/,PRJNA1046269.

## References

[B1] Armstrong A. Valverde A. Ramond J. B. Makhalanyane T. P. Jansson J. K. Hopkins D. W.. (2016). Temporal dynamics of hot desert microbial communities reveal structural and functional responses to water input. Sci. Rep. 6, 34434. doi: 10.1038/srep34434 27680878 PMC5041089

[B2] Bao S. D. (2020). Soil agrochemical analysis (Beijing: China Agriculture Press).

[B3] Bi B. Wang K. Zhang H. Wang Y. Fei H. Pan R.. (2021). Plants use rhizosphere metabolites to regulate soil microbial diversity. Land Degrad. Dev. 32, 5267–5280. doi: 10.1002/ldr.4107

[B4] Brown S. P. Grillo M. A. Podowski J. C. Heath K. D. (2020). Soil origin and plant genotype structure distinct microbiome compartments in the model legume *Medicago truncatula* . Microbiome. 8, 1–17. doi: 10.1186/s40168-020-00915-9 32988416 PMC7523075

[B5] Bulgarelli D. Rott M. Schlaeppi K. Ver Loren van Themaat E. Ahmadinejad N. Assenza F.. (2012). Revealing structure and assembly cues for *Arabidopsis* root-inhabiting bacterial microbiota. Nature 488, 91–95. doi: 10.1038/nature11336 22859207

[B6] Burrell A. L. Evans J. P. De Kauwe M. G. (2020). Anthropogenic climate change has driven over 5 million km^2^ of drylands towards desertification. Nat. Commun. 11, 3853. doi: 10.1038/s41467-020-17710-7 32737311 PMC7395722

[B7] Cavalcante A. L. A. Negreiros A. M. P. Tavares M. B. Barreto É. d. S. Armengol J. Sales Júnior R.. (2020). Characterization of five new *Monosporascus* species: Adaptation to environmental factors, pathogenicity to cucurbits, and sensitivity to fungicides. J. Fungi. 6, 169. doi: 10.3390/jof6030169 PMC7560037 32927599

[B8] Chandrasekaran M. Boughattas S. Hu S. Oh S. H. Sa T. (2014). A meta-analysis of arbuscular mycorrhizal effects on plants grown under salt stress. Mycorrhiza 24, 611–625. doi: 10.1007/s00572-014-0582-7 24770494

[B9] Chen X. He B. Ding C. Qi X. Li Y. Hu W. (2023). Diversity and functional distribution characteristics of myxobacterial communities in the rhizosphere of *Tamarix chinensis* Lour in Ebinur Lake Wetland, China. Microorganisms 11, 1924. doi: 10.3390/microorganisms11081924 37630484 PMC10459050

[B10] Chen K. H. Miadlikowska J. Molnár K. Arnold A. E. U’Ren J. M. Gaya E.. (2015). Phylogenetic analyses of eurotiomycetous endophytes reveal their close affinities to Chaetothyriales, Eurotiales, and a new order–Phaeomoniellales. Mol. Phylogenet. Evol. 85, 117–130. doi: 10.1016/j.ympev.2015.01.008 25701073

[B11] Chen P. Zhao M. Tang F. Hu Y. Peng X. Shen S. (2020). The effect of plant compartments on the *Broussonetia papyrifera*-associated fungal and bacterial communities. Appl. Microbiol. Biotechnol. 104, 3627–3641. doi: 10.1007/s00253-020-10466-6 32078018

[B12] Cheng X. Ping T. Li Z. Wang T. Han H. Epstein H. E. (2022). Effects of environmental factors on plant functional traits across different plant life forms in a temperate forest ecosystem. New For. 53, 125–142. doi: 10.1007/s11056-021-09847-0

[B13] Coyte K. Z. Schluter J. Foster K. R. (2015). The ecology of the microbiome: networks, competition, and stability. Science 350, 663–666. doi: 10.1126/science.aad2602 26542567

[B14] de Vries F. T. Griffiths R. I. Bailey M. Craig H. Girlanda M. Gweon H. S.. (2018). Soil bacterial networks are less stable under drought than fungal networks. Nat. Commun. 9, 3033. doi: 10.1038/s41467-018-05516-7 30072764 PMC6072794

[B15] de Zelicourt A. Al-Yousif M. Hirt H. (2013). Rhizosphere microbes as essential partners for plant stress tolerance. Mol. Plant 6, 242–245. doi: 10.1093/mp/sst028 23475999

[B16] Ding C. Hu W. Zhang X. Qi X. He B. Chen X. (2023). Composition and diversity of the fungal community in the rhizosphere soil of halophytic vegetation in Ebinur Lake wetland. Environ. Sci. Pollut. Res. 30, 86097–86109. doi: 10.1007/s11356-023-28221-5 37395876

[B17] Dong L. Li M. X. Li S. Yue L. X. Ali M. Han J. R.. (2024). Aridity drives the variability of desert soil microbiomes across north-western China. Sci. Total Environ. 907, 168048. doi: 10.1016/j.scitotenv.2023.168048 37890638

[B18] Du D. Jiao L. Wu X. Xue R. Wei M. Zhang P.. (2024). Drought determines the growth stability of different dominant conifer species in Central Asia. Glob. Planet. Change. 234, 104370. doi: 10.1016/j.gloplacha.2024.104370

[B19] Edwards J. Johnson C. Santos-Medellín C. Lurie E. Podishetty N. K. Bhatnagar S.. (2015). Structure, variation, and assembly of the root-associated microbiomes of rice. Proc. Natl. Acad. Sci. U.S.A. 112, E911–E920. doi: 10.1073/pnas.1414592112 25605935 PMC4345613

[B20] Fitzpatrick C. R. Copeland J. Wang P. W. Guttman D. S. Kotanen P. M. Johnson M. T. J. (2018). Assembly and ecological function of the root microbiome across angiosperm plant species. Proc. Natl. Acad. Sci. U.S.A. 115, E1157–E1165. doi: 10.1073/pnas.1717617115 29358405 PMC5819437

[B21] Foster K. R. Schluter J. Coyte K. Z. Rakoff-Nahoum S. (2017). The evolution of the host microbiome as an ecosystem on a leash. Nature 548, 43–51. doi: 10.1038/nature23292 28770836 PMC5749636

[B22] Gao L. Huang Y. Ma J. Jiang H. Li W. Li L. (2023). Diversity and roles of endophytic fungi in two halophytes. Microbiol. China. 50, 3357–3371. doi: 10.13344/j.microbiol.China.221032

[B23] Gao C. Xu L. Montoya L. Madera M. Hollingsworth J. Chen L.. (2022). Co-occurrence networks reveal more complexity than community composition in resistance and resilience of microbial communities. Nat. Commun. 13, 3867. doi: 10.1038/s41467-022-31343-y 35790741 PMC9256619

[B24] Gardes M. Bruns T. D. (1993). ITS primers with enhanced specificity for basidiomycetes - application to the identification of mycorrhizae and rusts. Mol. Ecol. 2, 113–118. doi: 10.1111/j.1365-294X.1993.tb00005.x 8180733

[B25] Guo B. Zhang L. Sun H. Gao M. Yu N. Zhang Q.. (2022). Microbial co-occurrence network topological properties link with reactor parameters and reveal importance of low-abundance genera. NPJ Biofilms Microbiomes 8, 3. doi: 10.1038/s41522-022-00273-4 35039527 PMC8764041

[B26] Harris J. (2009). Soil microbial communities and restoration ecology: Facilitators or followers? Science 325, 573–574. doi: 10.1126/science.1172975 19644111

[B27] He S. Hu W. Jin X. Han J. (2021). Soil bacterial community composition and diversity respond to soil environment in the Ebinur Lake Wetland. Arch. Microbiol. 203, 1175–1182. doi: 10.1007/s00203-020-02112-6 33226465

[B28] He Y. Hu W. Ma D. Lan H. Yang Y. Gao Y. (2017). Abundance and diversity of ammonia-oxidizing archaea and bacteria in the rhizosphere soil of three plants in the Ebinur Lake wetland. Can. J. Microbiol. 63, 573–582. doi: 10.1139/cjm-2016-0492 28249125

[B29] Hu R. Liu T. Zhang Y. Zheng R. Guo J. (2023). Leaf nutrient resorption of two life-form tree species in urban gardens and their response to soil nutrient availability. PeerJ 11, e15738. doi: 10.7717/peerj.15738 37483974 PMC10362843

[B30] Huang Y. Peng X. Ou G. Peng X. Gan L. Huang Y.. (2024). Effects of continuous cropping on fungal community structure succession in rhizosphere and non-rhizosphere soils of cassava. Guihaia 44, 1864–1877. doi: 10.11931/guihai.gxzw202311024

[B31] Husain H. Keitel C. Dijkstra F. A. (2024). Fungi are more important than bacteria for soil carbon loss through priming effects and carbon protection through aggregation. Appl. Soil Ecol. 195, 105245. doi: 10.1016/j.apsoil.2023.105245

[B32] Innerebner G. Knief C. Vorholt J. A. (2011). Protection of *Arabidopsis thaliana* against leaf-pathogenic *Pseudomonas syringae* by *Sphingomonas* strains in a controlled model system. Appl. Environ. Microbiol. 77, 3202–3210. doi: 10.1128/AEM.00133-11 21421777 PMC3126462

[B33] Isola D. Prigione V. P. Zucconi L. Varese G. C. Poli A. Turchetti B.. (2022). *Knufia obscura* sp. nov. and Knufia victoriae sp. nov., two new species from extreme environments. Int. J. Syst. Evol. Microbiol. 72, 5530. doi: 10.1099/ijsem.0.005530 36201346

[B34] Khuna S. Suwannarach N. Kumla J. Frisvad J. C. Matsui K. Nuangmek W.. (2021). Growth enhancement of *Arabidopsis* (*Arabidopsis thaliana*) and onion (*Allium cepa*) with inoculation of three newly identified mineral-solubilizing fungi in the genus *Aspergillus* section *Nigri* . Front. Microbiol. 12. doi: 10.3389/fmicb.2021.705896 PMC8397495 34456888

[B35] Lan G. Wei Y. Li Y. Wu Z. (2023). Diversity and assembly of root-associated microbiomes of rubber trees. Front. Plant Sci. 14. doi: 10.3389/fpls.2023.1136418 PMC10102524 37063173

[B36] Li M. Y. Wang J. L. Zhou Q. Zhang T. Mihray-Mutallip (2021). Analysis on the rhizosphere fungal community structure of four halophytes in Southern Xinjiang. Acta Ecol. Sin. 41, 8484–8495. doi: 10.5846/stxb202009032296

[B37] Li S. Wu F. (2018). Diversity and co-occurrence patterns of soil bacterial and fungal communities in seven intercropping systems. Front. Microbiol. 9. doi: 10.3389/fmicb.2018.01521 PMC6043683 30034385

[B38] Liang M. Liu X. Etienne R. S. Huang F. Wang Y. Yu S. (2015). Arbuscular mycorrhizal fungi counteract the Janzen-Connell effect of soil pathogens. Ecology 96, 562–574. doi: 10.1890/14-0871.1 26240876

[B39] Liu L. Ma L. Zhu M. Liu B. Liu X. Shi Y. (2023). Rhizosphere microbial community assembly and association networks strongly differ based on vegetation type at a local environment scale. Front. Microbiol. 14. doi: 10.3389/fmicb.2023.1129471 PMC10043216 36998396

[B40] López-García Á. Varela-Cervero S. Vasar M. Öpik M. Barea J. M. Azcón-Aguilar C. (2017). Plant traits determine the phylogenetic structure of arbuscular mycorrhizal fungal communities. Mol. Ecol. 26, 6948–6959. doi: 10.1111/mec.14403 29110362

[B41] Lundberg D. S. Lebeis S. L. Paredes S. H. Yourstone S. Gehring J. Malfatti S.. (2012). Defining the core *Arabidopsis thaliana* root microbiome. Nature 488, 86–90. doi: 10.1038/nature11237 22859206 PMC4074413

[B42] Luo J. Liu T. Diao F. Hao B. Zhang Z. Hou Y.. (2023). Shift in rhizospheric and endophytic microbial communities of dominant plants around Sunit Alkaline Lake. Sci. Total Environ. 867, 161503. doi: 10.1016/j.scitotenv.2023.161503 36634786

[B43] Maestre F. T. Delgado-Baquerizo M. Jeffries T. C. Eldridge D. J. Ochoa V. Gozalo B.. (2015). Increasing aridity reduces soil microbial diversity and abundance in global drylands. Proc. Natl. Acad. Sci. U S A 112, 15684–15689. doi: 10.1073/pnas.1516684112 26647180 PMC4697385

[B44] Manici L. M. Caputo F. De Sabata D. Fornasier F. (2024). The enzyme patterns of *Ascomycota* and *Basidiomycota* fungi reveal their different functions in soil. Appl. Soil Ecol. 196, 105323. doi: 10.1016/j.apsoil.2024.105323

[B45] Mengual C. Schoebitz M. Azcón R. Roldán A. (2014). Microbial inoculants and organic amendment improve plant establishment and soil rehabilitation under semiarid conditions. J. Environ. Manage. 134, 1–7. doi: 10.1016/j.jenvman.2014.01.008 24463051

[B46] Mercado-Blanco J. Abrantes I. Barra Caracciolo A. Bevivino A. Ciancio A. Grenni P.. (2018). Belowground microbiota and the health of tree crops. Front. Microbiol. 9. doi: 10.3389/fmicb.2018.01006 PMC5996133 29922245

[B47] Morriën E. Hannula S. E. Snoek B. L. Helmsing N. R. Zweers H. de Hollander M.. (2017). Soil networks become more connected and take up more carbon as nature restoration progresses. Nat. Commun. 8, 14349. doi: 10.1038/ncomms14349 28176768 PMC5309817

[B48] Müller M. E. H. Urban K. Köppen R. Siegel D. Korn U. Koch M. (2015). Mycotoxins as antagonistic or supporting agents in the interaction between phytopathogenic *Fusarium* and *Alternaria* fungi. World Mycotoxin J. 8, 311–322. doi: 10.3920/wmj2014.1747

[B49] Niu Y. Hu W. Zhou T. He B. Chen X. Li Y. (2022). Diversity of nirS and nirK denitrifying bacteria in rhizosphere and non-rhizosphere soils of halophytes in Ebinur Lake Wetland. Biotechnol. Biotechnol. Equip. 36, 209–219. doi: 10.1080/13102818.2022.2070030

[B50] Osés-Pedraza R. Torres-Díaz C. Lavín P. Retamales-Molina P. Atala C. Gallardo-Cerda J.. (2020). Root endophytic *Penicillium* promotes growth of Antarctic vascular plants by enhancing nitrogen mineralization. Extremophiles 24, 721–732. doi: 10.1007/s00792-020-01189-7 32699913

[B51] Ouyang S. Tian Y. Liu Q. Zhang L. Wang R. Xu X. (2016). Nitrogen competition between three dominant plant species and microbes in a temperate grassland. Plant Soil. 408, 121–132. doi: 10.1007/s11104-016-2904-3

[B52] Pan Y. Kang P. Hu J. Song N. (2021). Bacterial community demonstrates stronger network connectivity than fungal community in desert-grassland salt marsh. Sci. Total Environ. 798, 149118. doi: 10.1016/j.scitotenv.2021.149118 34332392

[B53] Peng M. He H. Wang X. Wang Z. Zhuang L. (2023). Comparison of network connectivity and environmental driving factors of root-associated fungal communities of desert ephemeral plants in two habitat soils. J. Environ. Manage. 332, 117375. doi: 10.1016/j.jenvman.2023.117375 36716547

[B54] Perez-Harguindeguy N. Díaz S. Garnier E. Lavorel S. Poorter H. Jaureguiberry P.. (2013). New handbook for standardised measurement of plant functional traits worldwide. Aust. J. Bot. 61, 167. doi: 10.1071/BT12225

[B55] Qi X. Chen T. Ding C. Chen X. He B. Hu W. (2023). Diversity of fungal communities in the rhizosphere soil of *Tamarix chinensis* in saline–alkaline wetland. Environ. Dev. Sustain. 27, 8693–8709. doi: 10.1007/s10668-023-04250-5

[B56] Qin K. Dong X. Jifon J. Leskovar D. I. (2019). Rhizosphere microbial biomass is affected by soil type, organic and water inputs in a bell pepper system. Appl. Soil Ecol. 138, 80–87. doi: 10.1016/j.apsoil.2019.02.024

[B57] Raju V. Gopal P. Suthari S. (2014). Environmental assessment of climate of a habitat through floristic life-form spectra, a case study of Warangal North forest division, Telangana, India. J. Nat. Sci. 2, 77–93.

[B58] Ren Y. Yu G. Shi C. Zhang L. Qin Q. Song X.. (2022). Majorbio Cloud: a one-stop, comprehensive bioinformatic platform for multiomics analyses. IMeta 1, e12. doi: 10.1002/imt2.12 38868573 PMC10989754

[B59] Ren C. Zhou Z. Guo Y. Yang G. Zhao F. Wei G.. (2021). Contrasting patterns of microbial community and enzyme activity between rhizosphere and bulk soil along an elevation gradient. Catena 196, 104921. doi: 10.1016/j.catena.2020.104921

[B60] Stein R. R. Bucci V. Toussaint N. C. Buffie C. G. Rätsch G. Pamer E. G.. (2013). Ecological modeling from time-series inference: insight into dynamics and stability of intestinal microbiota. PloS Comput. Biol. 9, e1003388. doi: 10.1371/journal.pcbi.1003388 24348232 PMC3861043

[B61] Tang Q. Zhu J. Chu M. Gu M. Sun J. Ghenijan O.. (2021). Community composition and distribution of endophytic fungi in *Salicornia europaea* from northern Xinjiang. J. Arid Land Res. Environ. 35, 137–143. doi: 10.13448/j.cnki.jalre.2021.139

[B62] Thepbandit W. Athinuwat D. (2024). Rhizosphere microorganisms supply availability of soil nutrients and induce plant defense. Microorganisms 12, 558. doi: 10.3390/microorganisms12030558 38543610 PMC10975764

[B63] Wagg C. Schlaeppi K. Banerjee S. Kuramae E. E. van der Heijden M. G. A. (2019). Fungal-bacterial diversity and microbiome complexity predict ecosystem functioning. Nat. Commun. 10, 4841. doi: 10.1038/s41467-019-12798-y 31649246 PMC6813331

[B64] Wang Q. Q. Lu J. H. Zhang J. Xu Y. Xu K. Zhang J. D.. (2022). Soil microbial community structure and its influencing factors in the original habitat of *Glycyrrhiza inflata* in different distribution areas. Acta Ecol. Sin. 42, 9780–9795. doi: 10.5846/stxb202108022098

[B65] Wang X. Zhang Y. Li J. Ding Y. Ma X. Zhang P.. (2025). Diversity and functional insights into endophytic fungi in halophytes from West Ordos Desert ecosystems. J. Fungi. 11, 30. doi: 10.3390/jof11010030 PMC11766765 39852449

[B66] White T. J. Bruns T. Lee S. (1990). “Amplification and direct sequencing of fungal ribosomal RNA genes for phylogenetics,” in PCR protocols: a guide to methods and applications (New York: Academic Press, Inc.), vol. 18. , 315–322.

[B67] Wu D. Jiang L. Li W. Wu Q. Lv G. (2023). Drivers of rhizosphere microbial differences in desert genus *Haloxylon* . Land Degrad. Dev. 34, 3513–3524. doi: 10.1002/ldr.4699

[B68] Xie T. Yu E. Kong Q. Lv G. (2009). Study on the halophytes in Aibi lake wetland nature reserve. J. Arid Land Resour. Environ. 23, 176–180. doi: 10.13448/j.cnki.jalre.2009.03.004

[B69] Xiong W. Li R. Ren Y. Liu C. Zhao Q. Wu H.. (2017). Distinct roles for soil fungal and bacterial communities associated with the suppression of vanilla *Fusarium* wilt disease. Soil Biol. Biochem. 107, 198–207. doi: 10.1016/j.soilbio.2017.01.010

[B70] Yang G. Jiang L. Li W. Li E. Lv G. (2023). Structural characteristics and assembly mechanisms of soil microbial communities under water-salt gradients in arid regions. Microorganisms 11, 1060. doi: 10.3390/microorganisms11041060 37110483 PMC10142023

[B71] Yeoh Y. K. Paungfoo-Lonhienne C. Dennis P. G. Robinson N. Ragan M. A. Schmidt S.. (2016). The core root microbiome of sugarcanes cultivated under varying nitrogen fertilizer application. Environ. Microbiol. 18, 1338–1351. doi: 10.1111/1462-2920.12925 26032777

[B72] Zhang X. Hu W. Jin X. Chen T. Niu Y. (2021). Diversity of soil nitrogen-fixing bacteria in the rhizosphere and non-rhizosphere soils of Ebinur Lake Wetland. Arch. Microbiol. 203, 3919–3932. doi: 10.1007/s00203-021-02363-x 34021386

[B73] Zhang S. Yuan M. Shi Z. Yang S. Zhang M. Sun L.. (2022). The variations of leaf δ13C and its response to environmental changes of arbuscular and ectomycorrhizal plants depend on life forms. Plants 11, 3236. doi: 10.3390/plants11233236 36501277 PMC9739095

[B74] Zhao Y. Li T. Shao P. Sun J. Xu W. Zhang Z. (2022). Structural characteristics of fungi communities in soil of different halophytes in the Yellow River Delta. Acta Bot. Boreal.-Occident. Sin. 42, 854–864. doi: 10.7606/i.issn.1000-4025.2022.05.0854

[B75] Zhao S. Liu J.-J. Banerjee S. Zhou N. Zhao Z.-Y. Zhang K.. (2018). Soil pH is equally important as salinity in shaping bacterial communities in saline soils under halophytic vegetation. Sci. Rep. 8, 4550. doi: 10.1038/s41598-018-22788-7 29540760 PMC5851986

[B76] Zhao D. Wei M. Wang X. Aqeel M. Ran J. Deng J. (2024). Morpho-physiological adaptations to drought stress in nitrogen-fixing and non-nitrogen-fixing plants. Front. Ecol. Evol. 12. doi: 10.3389/fevo.2024.1407882

[B77] Zheng W. Xue D. Li X. Deng Y. Rui J. Feng K.. (2017). The responses and adaptations of microbial communities to salinity in farmland soils: A molecular ecological network analysis. Appl. Soil Ecol. 120, 239–246. doi: 10.1016/j.apsoil.2017.08.019

[B78] Zhou Z. Wang G. Yu M. Gao G. Ding G. (2023). The leguminous *Hedysarum* shrubs effectively drive the diversity and structural composition of soil bacterial community through rhizocompartments in the process of desertification reversal. Land Degrad. Dev. 34, 4833–4846. doi: 10.1002/ldr.4812

[B79] Zhuang W. Yu X. Hu R. Luo Z. Liu X. Zheng X.. (2020). Diversity, function and assembly of mangrove root-associated microbial communities at a continuous fine-scale. NPJ Biofilms Microbiomes 6, 1–10. doi: 10.1038/s41522-020-00164-6 33184266 PMC7665043

[B80] Zuo Y. L. Hu Q. N. Qin L. Liu J. Q. He X. L. (2022). Species identity and combinations differ in their overall benefits to *Astragalus adsurgens* plants inoculated with single or multiple endophytic fungi under drought conditions. Front. Plant Sci. 13. doi: 10.3389/fpls.2022.933738 PMC9490189 36160950

