# Supplementary material for: Rhizocompartments drive the structure of root-associated fungal communities in halophytes with different life forms
Source: Front Plant Sci. 2025 Jun 3;16:1584398. doi: 10.3389/fpls.2025.1584398 (PMC12188457; doi:10.3389/fpls.2025.1584398)
Supplement: Supplementary file 1 [file SupplementaryFile1.docx]

Supplementary Material

## Supplementary Figures


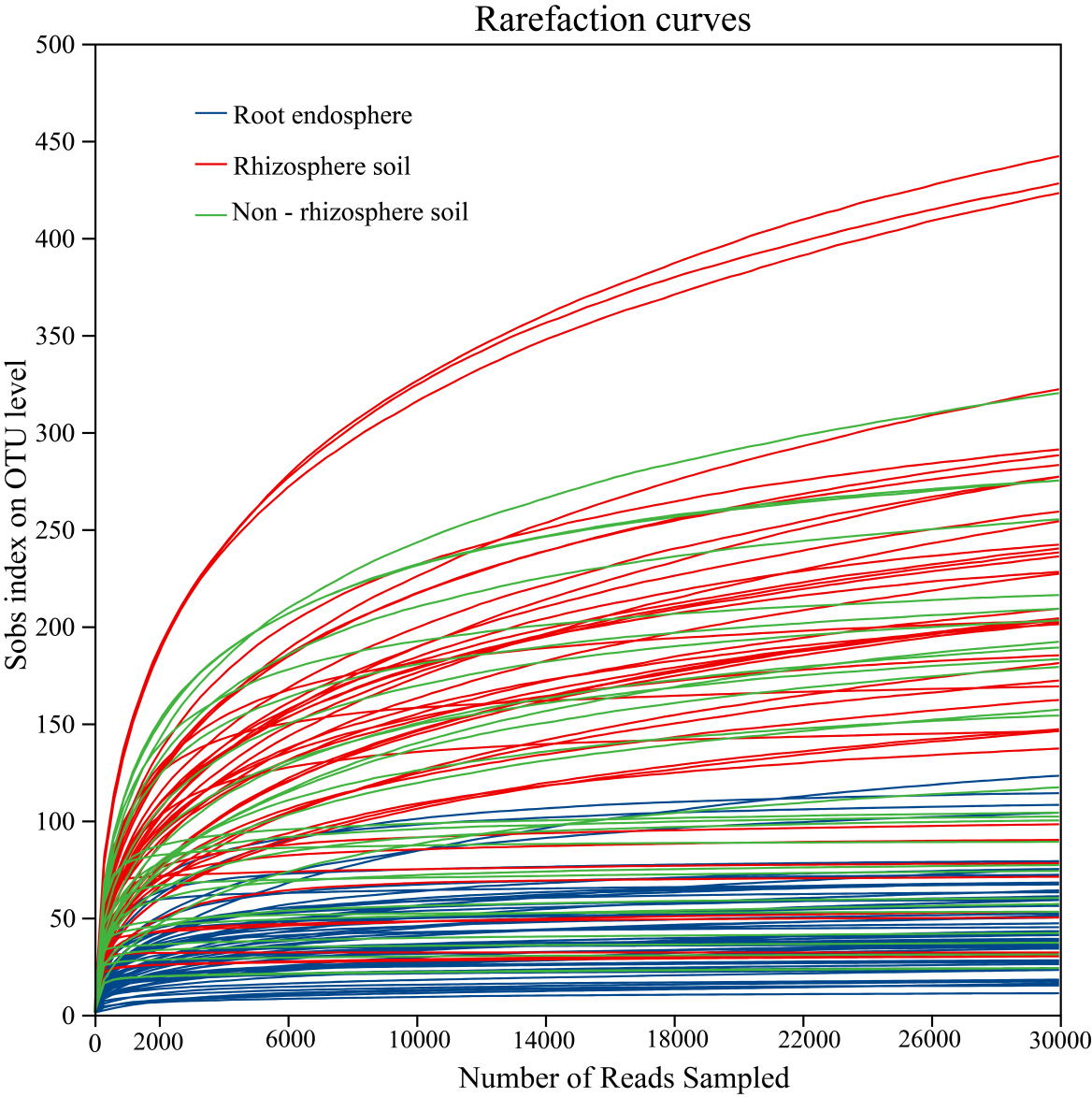


**Fig. S1.** The rarefaction curves of Sobs index at OTU level for three rhizocompartments. The horizontal axis represents the randomly sampled sequencing data volume; the vertical axis represents the Sobs index on OTU level.


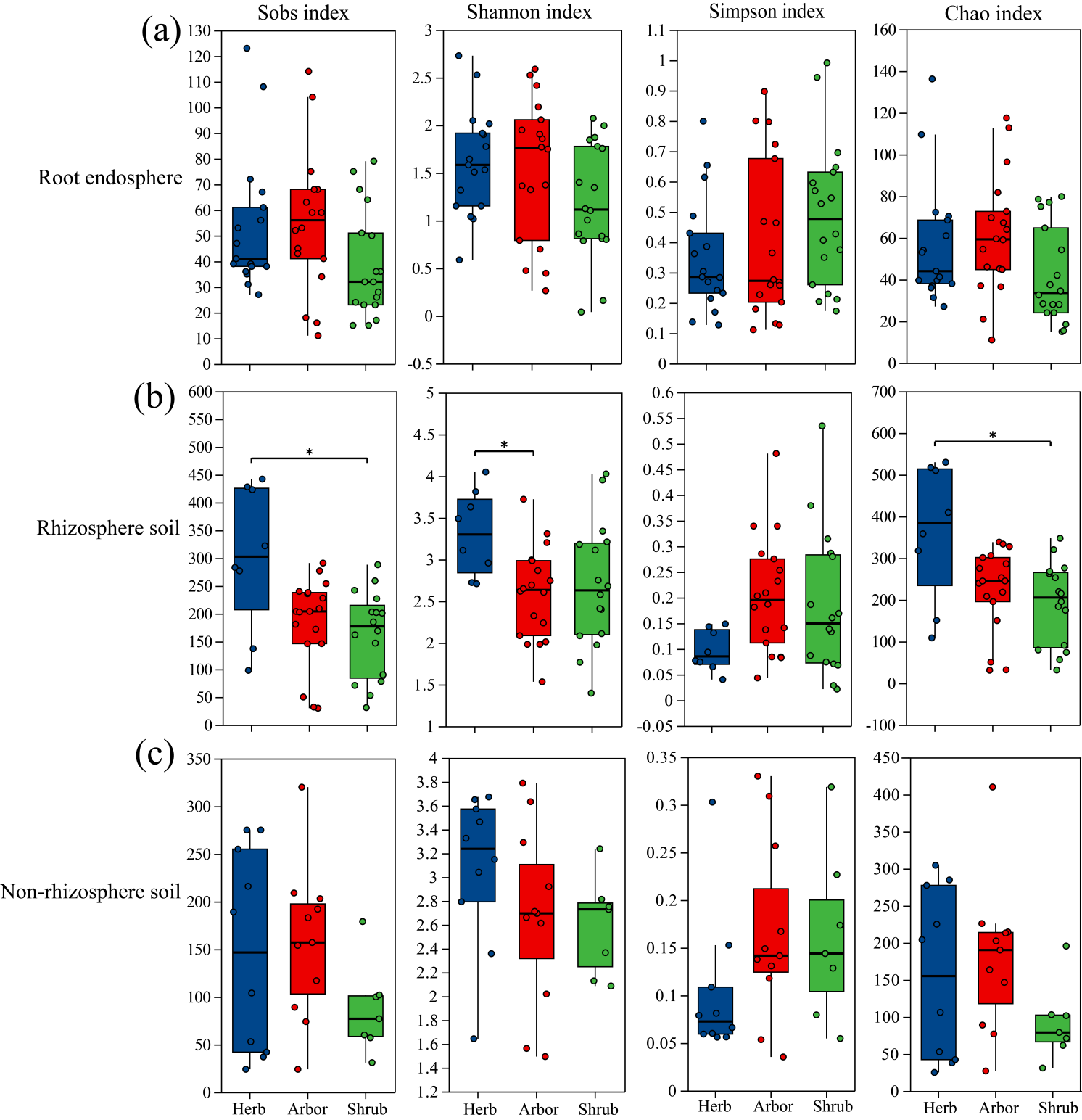


**Fig. S2.** The difference of α diversity index between different life form plants in three rhizocompartments. (a): four diversity indices of root endosphere; (b): Four diversity indices of rhizosphere soil; (c): Four diversity indices of non-rhizosphere soil. Significance determined via Kruskal-Wallis rank-sum test followed by Dunn’s test for multiple comparisons, * *P* ≤ 0.05, ** *P* ≤ 0.01, *** *P* ≤ 0.001.


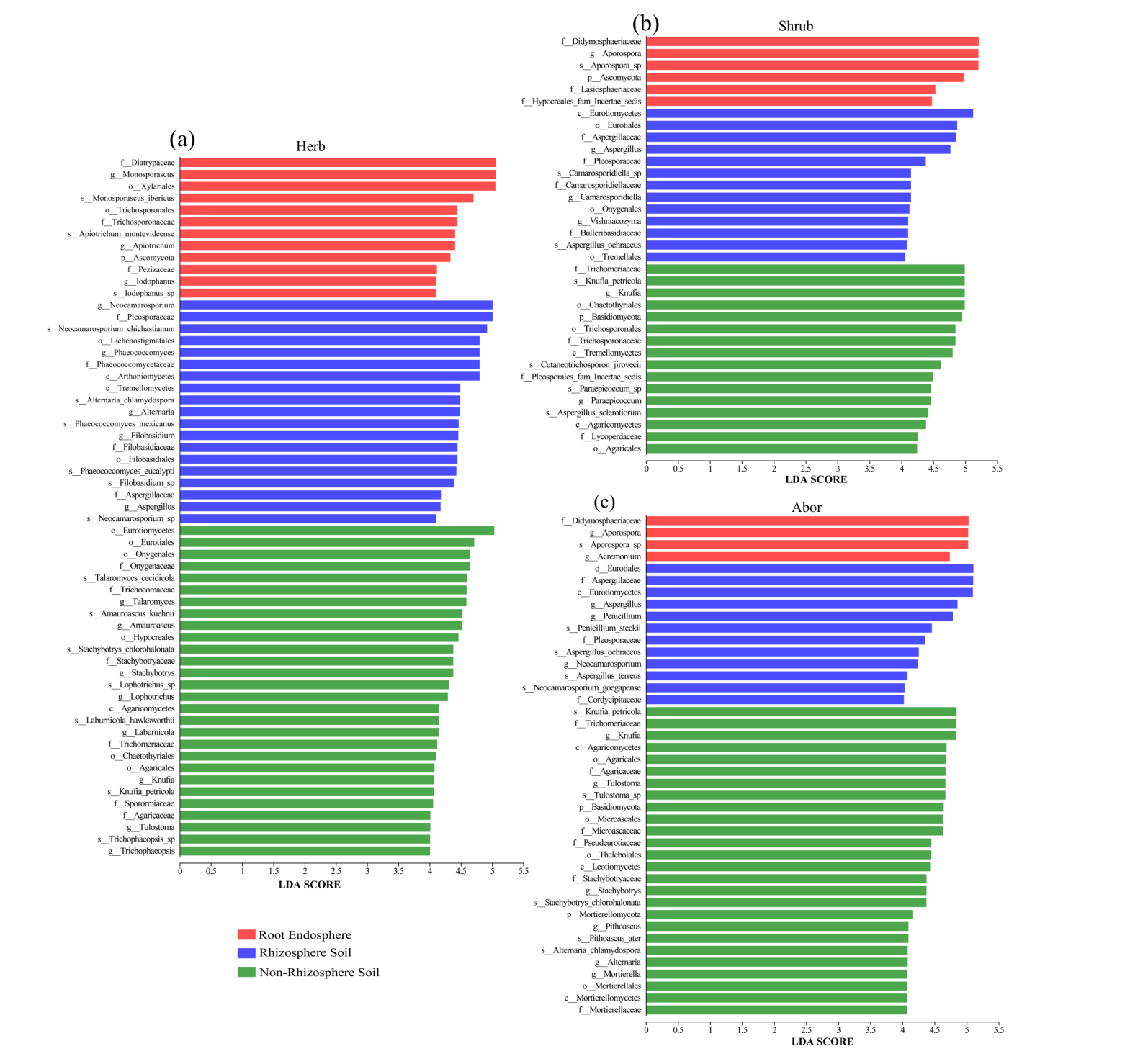


**Fig. S3.** LDA value distribution histogram of fungal community in three rhizocompartments of different life forms plants. (a) Herb; (b) Shrub; (c) Abor. Note: the figure only shows a significant difference in the LDA value > 4 fungal taxa.


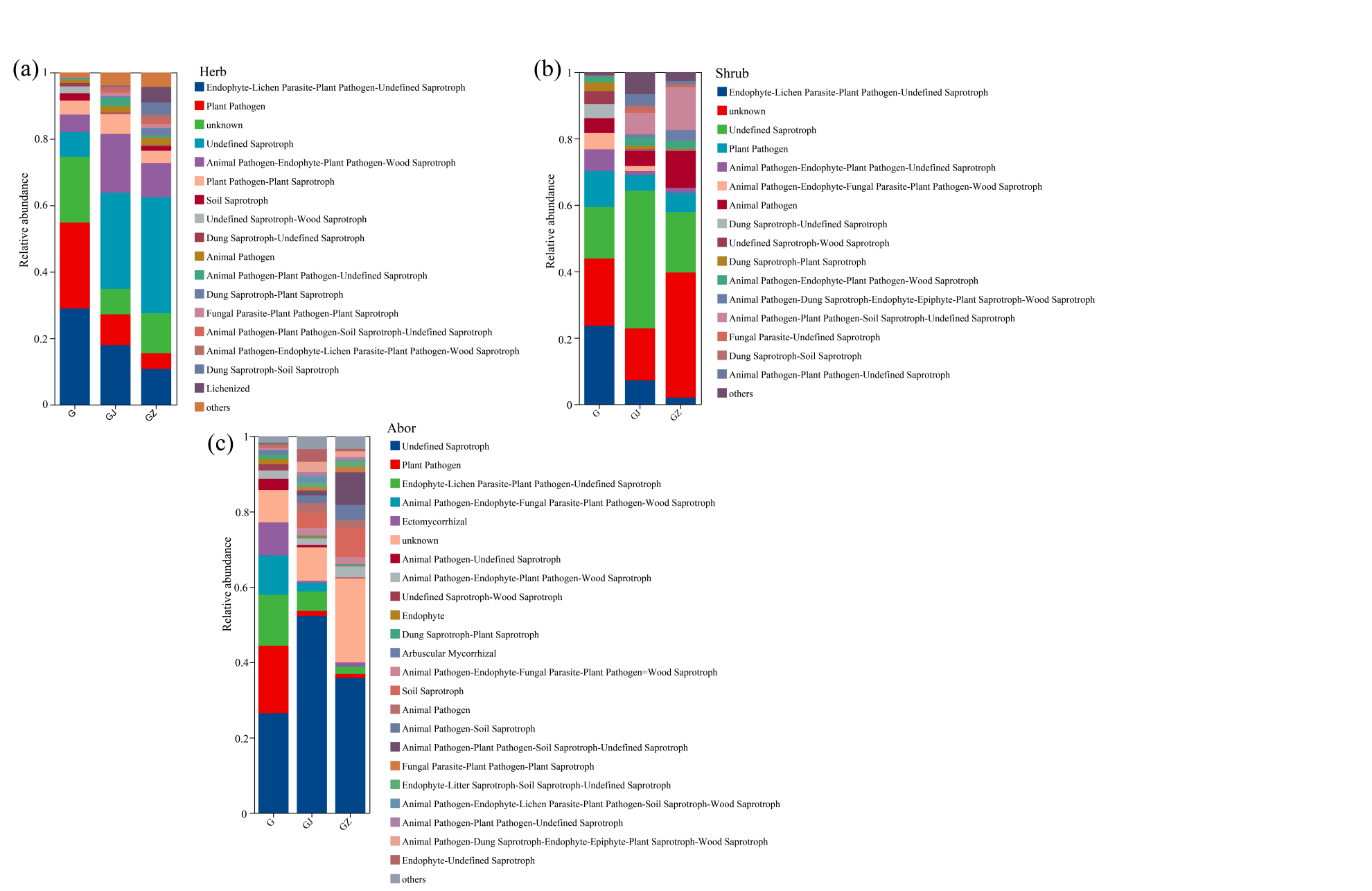


**Fig. S4.** Variations in composition of fungal functional groups inferred by FUNGuild. Note: (a): Herb; (b): Shrub; (c): Arbor. Combine functional guilds with an abundance of less than 0.01 into "other." G: root endosphere; GJ: rhizosphere soil; GZ: non-rhizosphere soil.

## Supplementary Table

**Table S1.** Main soil properties and their measurement methods

| Soil properties | Measurement method |
| --- | --- |
| Soil moisture content (SWC) | Weight method (fresh soil samples were dried at 105 ℃ to a constant weight) |
| Soil pH | Glass electrode method (with a water soil ratio of 5:1) |
| Soil total nitrogen (TN) | The Kjeldahl method |
| Soil available phosphorus (AP) | The sodium bicarbonate extraction and the molybdenum antimony colorimetric method |
| Soil ammonium nitrogen (AN) | The indophenol blue colorimetric method |
| Soil alkaline phosphatase (ALP) | The disodium phenyl phosphate colorimetric method |
| Soil total phosphorus (TP) | The molybdenum antimony colorimetric method |
| Soil nitrate nitrogen (NN) | The phenol disulfonic acid colorimetric method |
| Soil salt content (SC) | Electrical conductivity method |
| Soil organic carbon content (SOC) | The potassium dichromate dilution and heating method |
| Soil catalase activity (CAT) | The potassium permanganate titration method |
| Soil urease (Ure) | The sodium phenolate - sodium hypochlorite colorimetric method |

**Table S2.** Main plant properties and their measurement methods

| Plant properties | Measurement method |
| --- | --- |
| Root water content (GSWC) | The oven - drying method |
| Leaf water content (LSWC) | The oven - drying method |
| Leaf membrane stability index (MSI) | The conductivity method |
| Leaf pH (PpH) | Grind the green leaf tissue and extract it with distilled water, then filter and measure the pH using a pH meter (with a water to leaf sample volume ratio of 8:1) |
| Leaf total phosphorus (LTP) | The molybdenum antimony colorimetric method |
| Leaf organic carbon content (LSOC) | The potassium dichromate dilution and heating method |
| Leaf total nitrogen (LTN) | The Kjeldahl method |
| Root total nitrogen (GTN) | The Kjeldahl method |
| Root total phosphorus (GTP) | The molybdenum antimony colorimetric method |
| Root organic matter (GSOM) | The potassium dichromate dilution and heating method |
| Leaf organic matter (LSOM) | The potassium dichromate dilution and heating method |
| Root organic carbon content (GSOC) | The potassium dichromate dilution and heating method |

**Table S3.** Explanation of different grouping factors and physical and chemical factors on sample differences.

| Characteristics | SumsOfSqs | MeanSqs | F_Model | R^2^ | P_value | P_adjust |
| --- | --- | --- | --- | --- | --- | --- |
| Rhizocompartment | 4.16695 | 2.08347 | 5.05549 | 0.07771 | 0.001 | 0.00153 |
| Life form | 2.83502 | 1.41751 | 3.34934 | 0.05287 | 0.001 | 0.00153 |
| PpH | 1.43742 | 1.43742 | 3.33296 | 0.02681 | 0.001 | 0.00153 |
| GTP (g kg^-1^) | 1.06694 | 1.06694 | 2.4565 | 0.0199 | 0.001 | 0.00153 |
| GTN (g kg^-1^) | 0.89055 | 0.89055 | 2.04351 | 0.01661 | 0.001 | 0.00153 |
| LSOC (g kg^-1^) | 1.88181 | 1.88181 | 4.40085 | 0.03509 | 0.001 | 0.00153 |
| GSOC (g kg^-1^) | 1.01591 | 1.01591 | 2.33673 | 0.01895 | 0.001 | 0.00153 |
| SWC (%) | 1.39324 | 1.39324 | 3.22778 | 0.02598 | 0.001 | 0.00153 |
| SC (g kg^-1^) | 1.42209 | 1.42209 | 3.29644 | 0.02652 | 0.001 | 0.00153 |
| pH | 1.16201 | 1.16201 | 2.68023 | 0.02167 | 0.001 | 0.00153 |
| SOC (g kg^-1^) | 1.05602 | 1.05602 | 2.43085 | 0.01969 | 0.001 | 0.00153 |
| AN (mg kg^-1^) | 0.99378 | 0.99378 | 2.28486 | 0.01853 | 0.001 | 0.00153 |
| NN (mg kg^-1^) | 1.10894 | 1.10894 | 2.55522 | 0.02068 | 0.001 | 0.00153 |
| CAT (IU) | 1.08682 | 1.08682 | 2.5032 | 0.02027 | 0.001 | 0.00153 |
| ALP (IU) | 1.18099 | 1.18099 | 2.725 | 0.02202 | 0.001 | 0.00153 |
| LSWC (%) | 0.85932 | 0.85932 | 1.9707 | 0.01603 | 0.002 | 0.00248 |
| LTP (g kg^-1^) | 0.8502 | 0.8502 | 1.94942 | 0.01586 | 0.002 | 0.00248 |
| LTN (g kg^-1^) | 0.94385 | 0.94385 | 2.16802 | 0.0176 | 0.002 | 0.00248 |
| AP (mg kg^-1^) | 1.00046 | 1.00046 | 2.30051 | 0.01866 | 0.002 | 0.00248 |
| Ure (IU) | 0.85752 | 0.85752 | 1.96649 | 0.01599 | 0.003 | 0.00355 |
| TP (g kg^-1^) | 0.74849 | 0.74849 | 1.71292 | 0.01396 | 0.006 | 0.00678 |
| MSI (%) | 0.72646 | 0.72646 | 1.6618 | 0.01355 | 0.01 | 0.01083 |
| TN (g kg^-1^) | 0.72743 | 0.72743 | 1.66407 | 0.01357 | 0.016 | 0.01664 |
| GSWC (%) | 0.62777 | 0.62777 | 1.43337 | 0.01171 | 0.029 | 0.029 |

Note: The first column of the table represents grouping factors or physical and chemical factors, SumOfSqs represents total variance, MeanSqs represents average variance, F. Model represents F test value, and R^2^ represents the explanatory power of each factor to the sample difference. The greater the R^2^, the higher the explanatory power of the factor to the difference. *P* < 0.05 indicates that the reliability of this test is high. Rhizocompartment: three rhizocompartments, Life form: three life forms. SWC: water content, SC: salt content, pH: soil pH, SOC: organic carbon, AP: available phosphorus, TP: total phosphorus, TN: total nitrogen, AN: ammonium nitrogen, NN: nitrate nitrogen, CAT: catalase, ALP: alkaline phosphatase, Ure: urease, GSWC: root water content, LSWC: leaf water content, MSI: membrane stability index, PpH: leaf pH, LTP: leaf total phosphorus, LTN: leaf total nitrogen, GTP: root total phosphorus, GTN: root total nitrogen, LSOC: leaf organic carbon; GSOC: root organic carbon.

**Table S4.** Key topological characteristics of fungal communities in different root compartments of different life forms plants.

| Network properties | Herb | | | Shrub | | | Arbor | | |
| --- | --- | --- | --- | --- | --- | --- | --- | --- | --- |
| Network properties | Root endosphere | Rhizosphere soil | Non-rhizosphere soil | Root endosphere | Rhizosphere soil | Non-rhizosphere soil | Root endosphere | Rhizosphere soil | Non-rhizosphere soil |
| Number of nodes | 100 | 98 | 99 | 98 | 81 | 98 | 99 | 99 | 100 |
| Number of edges | 474 | 1168 | 680 | 544 | 169 | 98 | 99 | 394 | 496 |
| Average degree | 9.480 | 23.837 | 13.737 | 11.102 | 4.173 | 9.449 | 8.889 | 7.960 | 9.920 |
| Modularity | 0.478 | 0.209 | 0.579 | 0.419 | 0.595 | 0.636 | 0.512 | 0.457 | 0.496 |
| Number of communities | 5 | 4 | 5 | 5 | 7 | 9 | 6 | 7 | 6 |
| Average clustering coefficient | 0.507 | 0.609 | 0.666 | 0.542 | 0.393 | 0.730 | 0.421 | 0.395 | 0.505 |
| Average path length | 3.151 | 2.295 | 2.941 | 3.092 | 3.856 | 5.547 | 2.856 | 2.77 | 2.793 |
| Positive (%) | 94.73 | 86.73 | 91.47 | 97.98 | 98.82 | 89.63 | 91.82 | 87.82 | 91.73 |
| Negative (%) | 5.27 | 13.27 | 8.53 | 2.02 | 1.18 | 10.37 | 8.18 | 12.18 | 8.27 |

**Table S5.** Non-rhizosphere soil and plant physical and chemical properties of different life forms plants.

|  | | Herb | Shrub | Arbor |
| --- | --- | --- | --- | --- |
| Soil | SWC (%) | 8.39±7.72 a | 6.82±4.09 a | 9.21±6.19 a |
|  | SC (g kg^-1^) | 1.69±1.14 a | 1.52±0.45 a | 1.58±0.91 a |
|  | pH | 8.38±0.27 a | 8.34±0.23 a | 8.40±0.27 a |
|  | SOC (g kg^-1^) | 2.98±3.53 a | 3.71±3.04 a | 5.91±5.72 a |
|  | AP (mg kg^-1^) | 22.81±9.32 a | 19.50±5.06 a | 23.17±10.14 a |
|  | TP (g kg^-1^) | 0.35±0.13 a | 0.34±0.13 a | 0.31±0.12 a |
|  | TN (g kg^-1^) | 0.10±0.02 a | 0.11±0.03 a | 0.12±0.05 a |
|  | AN (mg kg^-1^) | 4.03±1.98 a | 4.03±2.44 a | 4.73±2.65 a |
|  | NN (mg kg^-1^) | 44.53±25.79 a | 68.72±44.29 a | 73.96±61.44 a |
|  | CAT (IU) | 0.04±0.01 a | 0.04±0.01 a | 0.03±0.01 a |
|  | ALP( IU) | 7.58±5.01 a | 10.64±7.56 ab | 21.07±21.51 b |
|  | Ure (IU) | 0.12±0.03 a | 0.14±0.06 a | 0.18±0.11 a |
| Plant | GSWC (%) | 20.84±6.62 a | 17.53±5.02 a | 17.47±5.51 a |
|  | LSWC (%) | 17.24±9.26 a | 14.35±8.78 a | 31.29±7.28 b |
|  | MSI (%) | 34.06±14.92 a | 36.52±6.68 a | 32.92±8.04 a |
|  | PpH | 7.29±0.30 b | 6.59±0.29 a | 6.39±0.25 a |
|  | LTP (g kg^-1^) | 0.10±0.05 a | 0.05±0.05 a | 0.07±0.12 a |
|  | LTN (g kg^-1^) | 1.39±0.32 a | 1.30±0.33 a | 1.13±0.29 a |
|  | GTP (g kg^-1^) | 0.05±0.05 a | 0.05±0.06 a | 0.02±0.02 a |
|  | GTN (g kg^-1^) | 0.72±0.25 a | 1.10±0.37 b | 0.94±0.38 ab |
|  | LSOC (g kg^-1^) | 250.83±20.34 a | 395.21±44.65 b | 422.47±91.48 b |
|  | GSOC (g kg^-1^) | 432.01±43.59 b | 367.99±60.45 a | 361.38±70.68 a |

Note: Values are the means ± standard deviation. In rows, different lowercase letters indicate significant differences (*P* < 0.05). SWC: water content, SC: salt content, pH: soil pH, SOC: organic carbon, AP: available phosphorus, TP: total phosphorus, TN: total nitrogen, AN: ammonium nitrogen, NN: nitrate nitrogen, CAT: catalase, ALP: alkaline phosphatase, Ure: urease, GSWC: root water content, LSWC: leaf water content, MSI: membrane stability index, PpH: leaf pH, LTP: leaf total phosphorus, LTN: leaf total nitrogen, GTP: root total phosphorus, GTN: root total nitrogen, LSOC: leaf organic carbon; GSOC: root organic carbon.
